# Supplementary material for: Metabolite profile of Nectandra oppositifolia Nees & Mart. and assessment of antitrypanosomal activity of bioactive compounds through efficiency analyses
Source: PLoS One. 2021 Feb 25;16(2):e0247334. doi: 10.1371/journal.pone.0247334 (PMC7906415; doi:10.1371/journal.pone.0247334)
Supplement: S1 File — (DOCX) [file pone.0247334.s001.docx]

**Metabolite profile of *Nectandra oppositifolia* and assessment of the antitrypanosomal activity of bioactive compounds through efficiency analyses**

Geanne A. Alves Conserva^1¶^, Luis M. Quirós-Guerrero^2,3¶^, Thais A. Costa-Silva^1^, Laurence Marcourt^2,3^, Erika G. Pinto^4^, Andre G. Tempone^5^, João Paulo S. Fernandes^6^, Jean-Luc Wolfender^2,3*^, Emerson F. Queiroz^2,3*^, João Henrique G. Lago^1*^

^1^ Center of Natural Sciences and Humanities, Federal University of ABC, Santo Andre, Sao Paulo, Brazil.

^2^ School of Pharmaceutical Sciences, University of Geneva, Geneva, Switzerland.

^3^ Institute of Pharmaceutical Sciences of Western Switzerland, University of Geneva, Geneva, Switzerland

^4^ Drug Discovery Unit, School of Life Sciences, University of Dundee, Dundee, United Kingdom.

^5^ Centre for Parasitology and Mycology, Adolfo Lutz Institute, São Paulo, Brazil.

^6^ Department of Pharmaceutical Sciences, Federal University of São Paulo, Diadema, São Paulo, Brazil.

^¶^ These authors contributed equally to this work.

* Corresponding authors

E-mails: [jean-luc.wolfender@unige.ch](mailto:jean-luc.wolfender@unige.ch) (J.L.W.), [emerson.ferreira@unige.ch](mailto:emerson.ferreira@unige.ch)

(E.F.Q.), and [joao.lago@ufabc.edu.br](mailto:joao.lago@ufabc.edu.br) (J.H.G.L.)

SPECTROSCOPIC AND SPECTROMETRIC DATA OF COMPOUNDS **1 – 12**

*Ethyl (R)-pyroglutamate (****1****)*

White amorphous solid. [α]_D_^25^ = - 87.6 (*c* 0.0004, MeOH). ESI-HRMS *m/z* 158.0811 [M + H]^+^ (calcd. for C_7_H_12_NO_3_, 158.0817). ^1^H NMR (CD_3_OD, 600 MHz) δ/ppm: 1.28 (3H, t, *J* = 7.1 Hz, H-8), 2.15 (1H, dddd, *J* = 13.3, 9.7, 5.5, and 4.4 Hz, H-4a), 2.34 (2H, m, H-3), 2.48 (1H, m, H-4b), 4.21 (2H, q, *J* = 7.1 Hz, H-7), 4.28 (1H, dd, *J* = 9.1 and 4.4 Hz, H-5). ^13^C NMR (CD_3_OD, 151 MHz) δ/ppm: 14.2 (C-8), 25.6 (C-4), 30.1 (C-3), 56.9 (C-5), 62.3 (C-7), 173.8 (C-6), 180.9 (C-2).

*Indole-3-aldehyde (****2****)*

**

White amorphous solid. ESI-HRMS *m/z* 146.0604 [M + H]^+^ (calcd. for C_9_H_8_NO, 146.0605). ^1^H NMR (CD_3_OD, 600 MHz) δ/ppm: 7.24 (1H, td, *J* = 7.5 and 1.1 Hz, H-5), 7.28 (1H, td, *J* = 7.5 and 1.1 Hz, H-6), 7.48 (1H, dt, *J* = 7.5 and 1.1 Hz, H-4), 8.10 (1H, s, H-2), 8.16 (1H, dt, *J* = 7.5 and 1.1 Hz, H-7), 9.89 (1H, s, H-8). ^13^C NMR (CD_3_OD, 151 MHz) δ/ppm: 112.8 (C-4), 119.8 (C-3), 122.1 (C-7), 123.3 (C-5), 124.7 (C-6), 125.5 (C-3a), 139.3 (C-7a), 139.9 (C-2), 187.5 (C-8).

*(+)-(S)-Abscisic acid (****3****)*

White amorphous solid. [α]_D_^25^ = + 77.6 (*c* 0.0002, CH_2_Cl_2_). ESI-HRMS *m/z* 265.1435 [M + H]^+^ (calcd. for C_15_H_21_O_4_, 265.1439). ^1^H NMR (CDCl_3_, 600 MHz) δ/ppm: 1.02 (3H, s, H-8'), 1.11 (3H, s, H-9'), 1.92 (3H, d, *J* = 1.4 Hz, H-7'), 2.04 (3H, d, *J* = 1.3 Hz, H-6), 2.30 (1H, d, *J* = 17.1 Hz, H-5'b), 2.48 (1H, d, *J* = 17.1 Hz, H-5'a), 5.79 (1H, s, H-2), 5.94 (1H, p, *J* = 1.4 Hz, H-3'), 6.19 (1H, d, *J* = 16.1 Hz, H-5), 7.83 (1H, d, *J* = 16.1 Hz, H-4). ^13^C NMR (CDCl_3_, 151 MHz) δ/ppm: 18.8 (C-7’), 21.3 (C-6), 23.0 (C-9’), 24.3 (C-8’), 41.5 (C-6’), 49.7 (C-5’), 79.7 (C-1’), 116.9 (C-2), 127.1 (C-3’), 128.0 (C-4), 136.9 (C-5), 151.3 (C-3), 162.2 (C-2’), 197.7 (C-4’).

*2*Z*-(2,4-Dihydroxy-2,6,6-trimethylcyclohexylidene) acetic acid (****4****)*

White amorphous solid. [α]_D_^25^ = - 55.7 (*c* 0.0013, MeOH). ESI-HRMS *m/z* 197.1178 [M - H]^-^ (calcd. for C_11_H_17_O_3_, 197.1177). ^1^H NMR (CD_3_OD, 600 MHz) δ/ppm: 1.28 (3H, s, H-11), 1.47 (3H, s, H-10), 1.53 (1H, dd, *J* = 14.4 and 2.6 Hz, H-5''), 1.75 (1H, dd, *J* = 13.6 and 2.6 Hz, H-3''), 1.76 (3H, s, H-9), 1.99 (1H, dt, *J* = 14.4 and 2.6 Hz, H-5'), 2.42 (1H, dt, *J* = 13.6 and 2.6 Hz, H-3'), 4.21 (1H, d, *J* = 2.6 Hz, H-4), 5.75 (1H, s, 7). ^13^C NMR (CD_3_OD, 151 MHz) δ/ppm: 27.0 (C-10), 27.4 (C-9), 31.0 (C-11), 37.2 (C-6), 46.4 (C-3), 48.0 (C-5), 67.3 (C-4), 89.0 (C-2), 113.3 (C-7), 174.4 (C-8), 185.7 (C-1).

*Azelaic acid (****5****)*

White amorphous solid. ESI-HRMS *m/z* 187.0975 [M – H]^-^ (calcd. for C_9_H_15_O_4_, 187.0970). ^1^H NMR (CD_3_OD, 600 MHz) δ/ppm: 1.35 (6H, m, H-4, H-5, H-6), 1.60 (4H, d, *J* = 7.5 Hz, H-3, H-7), 2.25 (4H, t, *J* = 7.5 Hz, H-2, H-8). ^13^C NMR (CD_3_OD, 151 MHz) δ/ppm: 26.0 (C-3, C-7), 29.8 (C-4, C-5, C-6), 35.5 (C-2, C-8), 178.5 (C-1, C-9).

*Vanillic acid* *(****6****)*

White amorphous solid. ESI-HRMS *m/z* 167.0349 [M – H]^-^ (calcd for C_8_H_7_O_4,_ 167.0344). ^1^H NMR (CD_3_OD, 600 MHz) δ/ppm: 3.89 (3H, s, H-8), 6.83 (1H, d, *J* = 8.1 Hz, H-5), 7.55 (1H, dd, *J* = 8.1, 1.9 Hz, H-6), 7.56 (1H, d, *J* = 1.9 Hz, H-2). ^13^C NMR (CD_3_OD, 151 MHz) δ/ppm: 56.4 (C-8), 113.8 (C-2), 115.8 (C-5), 123.7 (C-1), 125.2 (C-6), 148.6 (C-3), 152.5 (C-4), 170.5 (C-7).

*Ethyl protocatechuate (****7****)*

White amorphous solid. ESI-HRMS *m/z* 181.0501 [M - H]^-^ (calcd. for C_9_H_9_O_4_, 181.0500). ^1^H NMR (CD_3_OD, 600 MHz) δ/ppm: 1.35 (3H, t, *J* = 7.1 Hz, H-3’), 4.29 (2H, q, *J* = 7.1 Hz, H-2’), 6.79 (1H, d, *J* = 8.3 Hz, H-5), 7.41 (1H, dd, *J* = 8.3 and 2.1 Hz, H-6), 7.42 (1H, d, *J* = 2.1 Hz, H-2); ^13^C NMR (CD_3_OD, 151 MHz) δ/ppm: 14.3 (C-3’), 61.3 (C-2’), 115.5 (C-5), 117.1 (C-2), 122.5 (C-1), 123.2 (C-6), 145.9 (C-3), 151.4 (C-4), 168.1 (C-1).

*Scopoletin (****8****)*

Yellow amorphous solid. ESI-HRMS *m/z* 193.0494 [M + H]^+^ (calcd. for C_10_H_9_O_4_, 193.0500). ^1^H NMR (CD_3_OD, 600 MHz) δ/ppm: 3.91 (3H, s, H-10), 6.21 (1H, d, *J* = 9.4 Hz, H-2), 6.78 (1H, s, H-8), 7.12 (1H, s, H-5), 7.86 (1H, d, *J* = 9.4 Hz, H-3). ^13^C NMR (CD_3_OD, 151 MHz) δ/ppm: 56.6 (C-10), 103.8 (C-8), 109.8 (C-5),112.3 (C-2, C-4), 146.0 (C-3), 147.0 (C-6), 151.3 (C-9), 153.0 (C-7), 169.3 (C-1).

(*-)-Evofolin B (****9****)*

White amorphous solid. [α]_D_^25^ = - 8.8 (*c* 0.003, MeOH). ESI-HRMS *m/z* 319.1177 [M + H]^+^ (calcd. for C_17_H_19_O_6,_ 319.1181). ^1^H NMR (CD_3_OD) δ/ppm: 7.62 (dd, *J* = 8.3 and 2.0 Hz, H-6), 7.56 (d, *J* = 2.0 Hz, H-2), 6.90 (d, *J* = 2.0 Hz, H-2’), 6.81 (d, *J* = 8.3 Hz, H-5), 6.77 (dd, *J* = 8.1 and 2.0 Hz, H-6’), 6.73 (d, *J* = 8.1 Hz, H-5’), 4.77 (dd, *J* = 8.8 and 5.2 Hz, H-α), 4.27 (dd, *J* = 10.6 and 8.8 Hz, H-βb), 3.86 (s, MeO-3’), 3.82 (s, MeO-3), 3.71 (dd, *J* = 10.6 and 5.2 Hz, H-βa). ^13^C NMR (CD_3_OD) δ/ppm: 199.5 (C-7), 152.9 (C-4), 148.9 (C-3’), 148.7 (C-3), 146.8 (C-4’), 130.1 (C-1), 129.7 (C-1’), 124.9 (C-6), 121.9 (C-6’), 116.3 (C-5’), 115.5 (C-5), 112.5 (C-2’), 112.1 (C-2), 65.2 (C-β), 56.1 (MeO-3/3’), 55.9 (C-α).

*Moupinamide (****10****)*


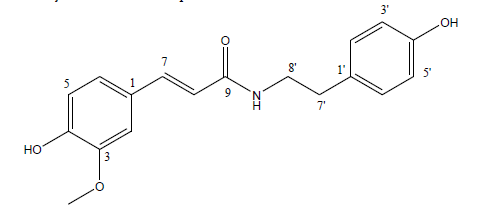


White amorphous solid. ESI-HRMS *m/z* 314.1383 [M + H]^+^ (calcd. for C_18_H_20_NO_4_, 314.1392). ^1^H NMR (CD_3_OD, 600 MHz) δ/ppm: 2.76 (t, *J* = 7.5 Hz, H-7’), 3.47 (t, *J* = 7.5 Hz, H-8’), 3.88 (s, OCH_3_), 6.41 (d, *J* = 15.5 Hz, H-8), 6.72 (d, *J* = 8.4 Hz, H-73/H-5’), 6.80 (d, *J* = 8.1 Hz, H-5), 7.03 (dd, *J* = 8.1 and 1.8 Hz, H-6), 7.06 (d, *J* = 8.4 Hz, H-2’/H-6’), 7.12 (d, *J* = 1.2 Hz, H-2), 7.44 (d, *J* = 15.5 Hz, H-7). ^13^C NMR (CD_3_OD, 151 MHz) δ/ppm: 35.6 (C-7’), 42.3 (C-8’), 56.3 (OCH_3_), 111.5 (C-2), 116.2 (C-3’/C-5’), 116.4 (C-5), 118.6 (C-8), 123.2 (C-6), 128.2 (C-1), 130.6 (C-2’/C-6’), 131.5 (C-1’), 142.0 (C-7), 149.3 (C-3), 149.8 (C-4), 156.9 (C-4’), 169.2 (C-9).

*Verrucosin (****11****)*

Pale yellow amorphous solid. [α]_D_^25^ = + 49.0 (*c* 0.0008, MeOH). ESI-HRMS *m/z* 345.1698 [M + H]^+^ (calcd. for C_20_H_25_O_5_, 345.1702). ^1^H NMR (CD_3_OD, 600 MHz) δ/ppm: 0.67 (d, *J* = 7.0 Hz, H-9’), 1.03 (d, *J* = 6.5 Hz, H-9), 1.80 (m, H-8), 2.26 (m, H-8’), 3.84 (s, 3’-OCH_3_), 3.88 (s, 3-OCH_3_), 4.37 (d, *J* = 9.5 Hz, H-7’), 5.11 (d, *J* = 8.9, H-7), 6.80 (m, H-6’), 6.83 (m, H-5), 6.93 (d, *J* = 1.8 Hz, H-2’), 6.96 (dd, *J* = 8.1 and 1.8, H-6), 6.99 (dd, *J* = 8.1 Hz, H-5’), 7.08 (d, *J* = 1.8 Hz, H-2). ^13^C NMR (CD_3_OD, 151 MHz) δ/ppm: 14.8 (C-9), 15.2 (C-9’), 47.2 (C-8’), 49.1 (C-8), 56.4 (3-OCH_3_/3’-OCH_3_), 84.6 (C-7’), 89.0 (C-7), 111.8 (C-2’), 112.0 (C-2), 115.8 (C-5’), 116.2 (C-5), 120.9 (C-6), 120.7 (C-6’), 133.3 (C-1), 134.3 (C-1’), 147.6 (C-4’), 147.8 (C-4), 149.1 (C-3), 149.2 (C-3’).

*Nectandrin B (****12****)*

Pale yellow amorphous solid. [α]_D_^25^ = 0 (*c* 0.0014, MeOH). ESI-HRMS *m/z* 343.1555 [M – H]^-^ (calcd. for C_20_H_23_O_5_, 343.1545). ^1^H NMR (CD_3_OD, 600 MHz) δ/ppm: 1.04 (d, *J* = 6.6 Hz, H-9/H-9’), 2.35 (m, H-8/H-8’), 3.85 (s, 3-OCH_3_/3’-OCH_3_), 4.48 (d, *J* = 6.5, H-7/H-7’), 6.80 (d, *J* = 8.0 Hz, H-5/H-5’), 6.89 (dd, *J* = 8.0 and 1.8 Hz, H-4/H-4’), 7.02 (d, *J* = 1.8 Hz, H-2/H-2’). ^13^C NMR (CD_3_OD, 151 MHz) δ/ppm: 11.7 (C-9/C-9’), 44.2 (C-8/C-8’), 55.0 (3-OCH_3_/3’-OCH_3_), 87.6 (C-7/C-7’), 109.8 (C-2/C-2’), 114.6 (C-5/C-5’), 119.0 (C-6/C-6’), 133.3 (C-1/C-1’), 145.9 (C-4/C-4’), 147.6 (C-3/C-3’).

**S1 Fig**. ^1^H-NMR spectrum of compound **1** (600 MHz, CD_3_OD)


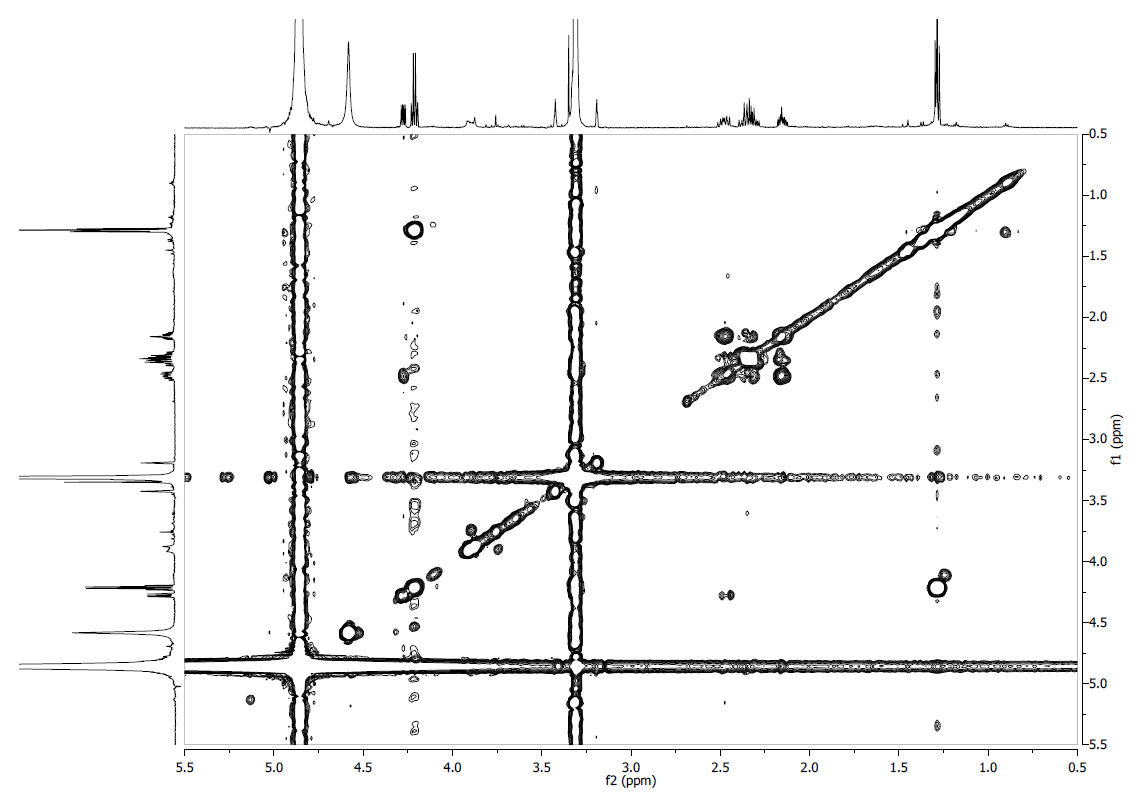


**S2 Fig**. COSY-NMR spectrum of compound **1** (CD_3_OD)

******

**S3 Fig.** Edited-HSQC-NMR spectrum of compound **1** (CD_3_OD)

**S4 Fig.** HMBC-NMR spectrum of compound **1** (CD_3_OD)


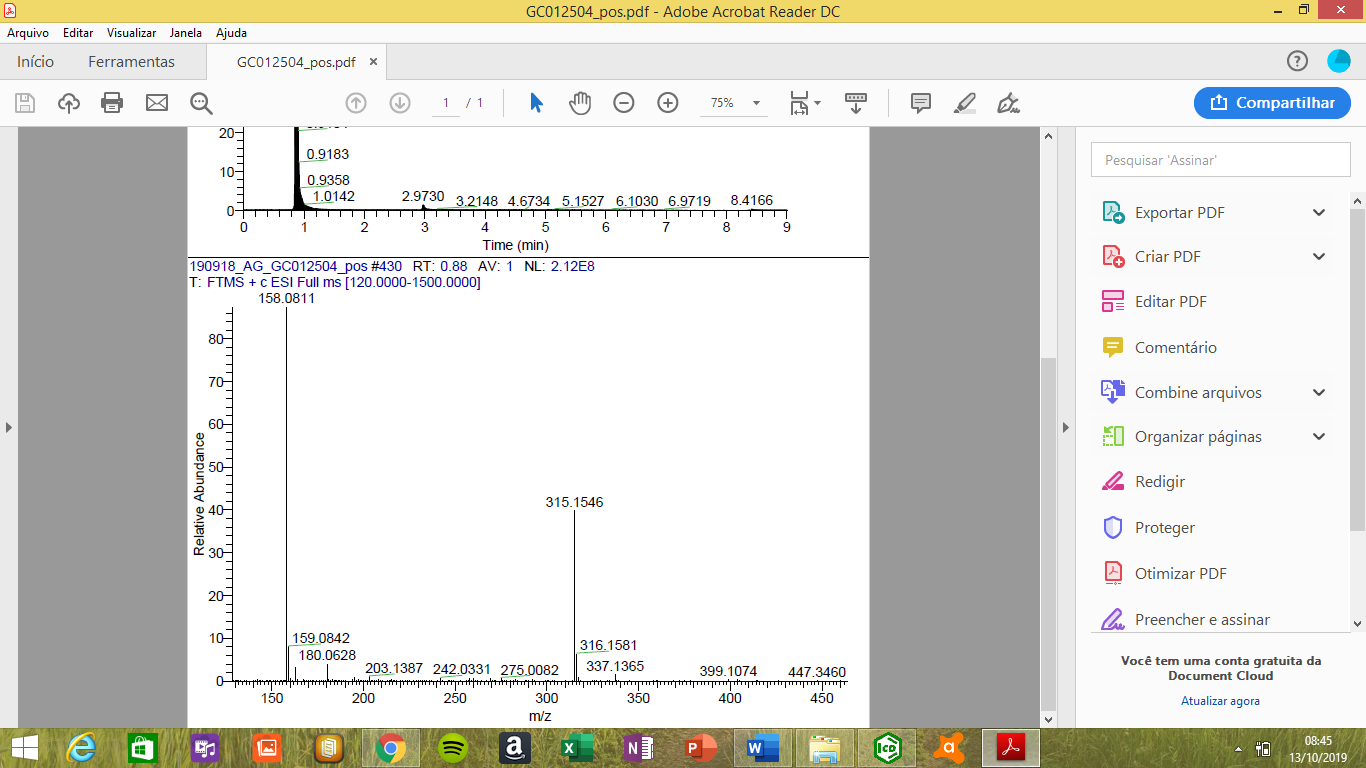


**S5 Fig.** HR-ESI-MS spectrum (positive mode) of compound **1**

**S6 Fig.** ^1^H-NMR spectrum of compound **2** (600 MHz, CD_3_OD)


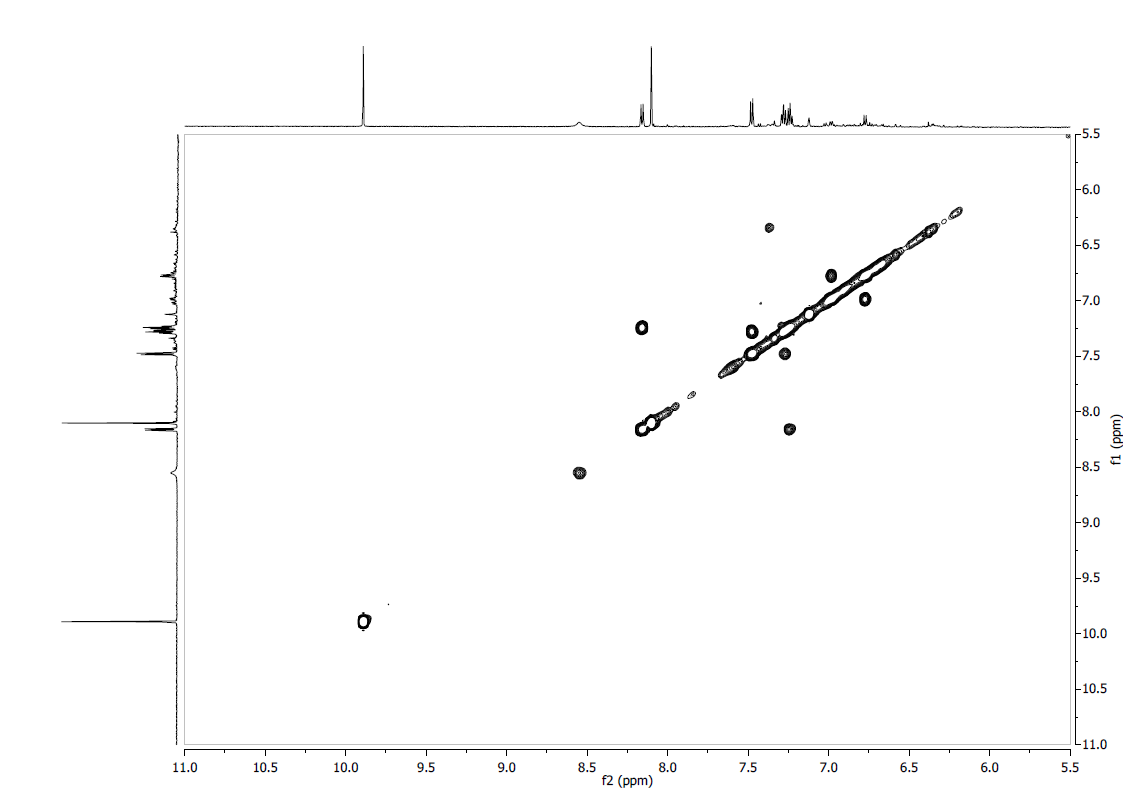


**S7 Fig.** COSY-NMR spectrum of compound **2** (CD_3_OD)


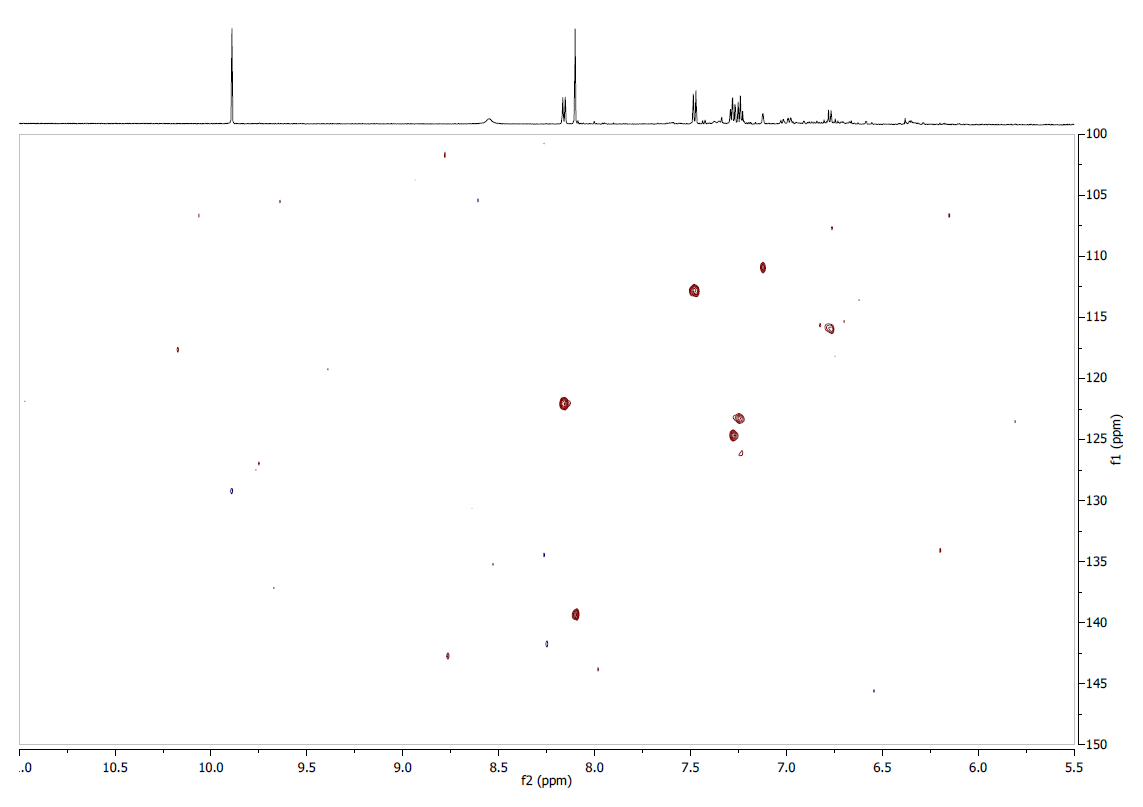


**S8 Fig.** Edited- HSQC-NMR spectrum of compound **2** (CD_3_OD)

**

**S9 Fig.** HMBC-NMR spectrum of compound **2** (CD_3_OD)

*
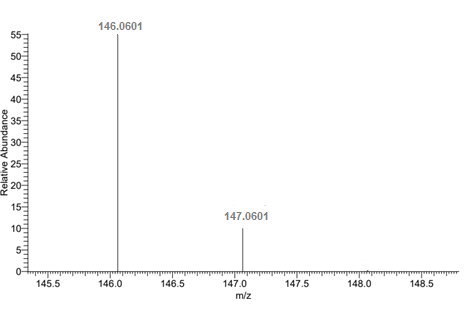
*

**10 Fig.** HR-ESI-MS spectrum (positive mode) of compound **2**

**S11 Fig.** ^1^H-NMR spectrum of compound **3** (600 MHz, CDCl_3_)


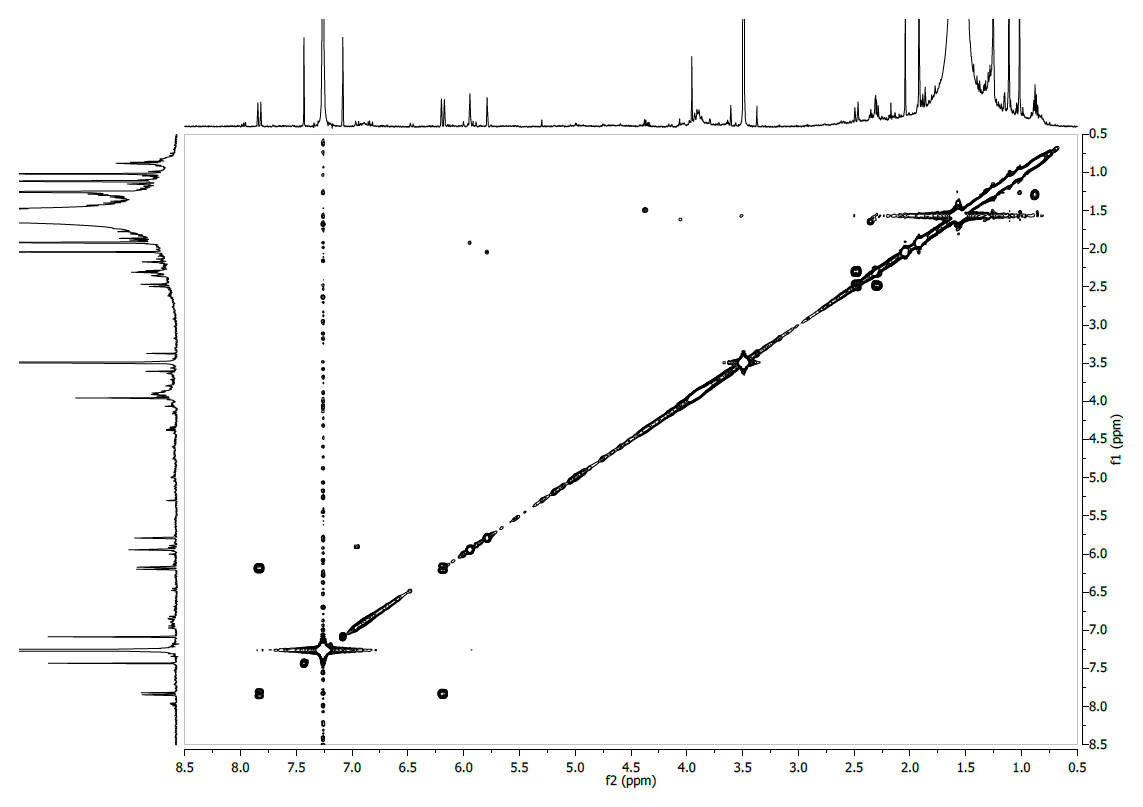


**S12 Fig.** COSY-NMR spectrum of compound **3** (CDCl_3_)

******

**S13 Fig.** Edited-HSQC-NMR spectrum of compound **3** (CDCl_3_)


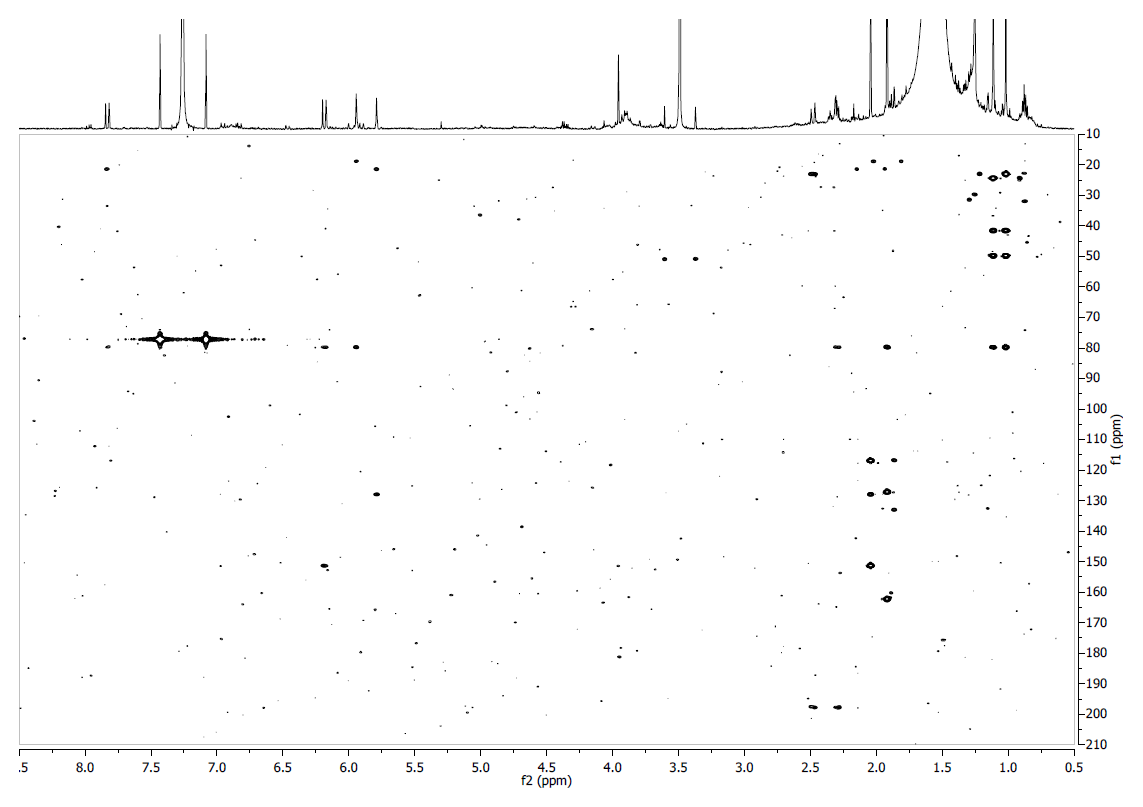


**S14 Fig.** HMBC-NMR spectrum of compound **3** (CDCl_3_)


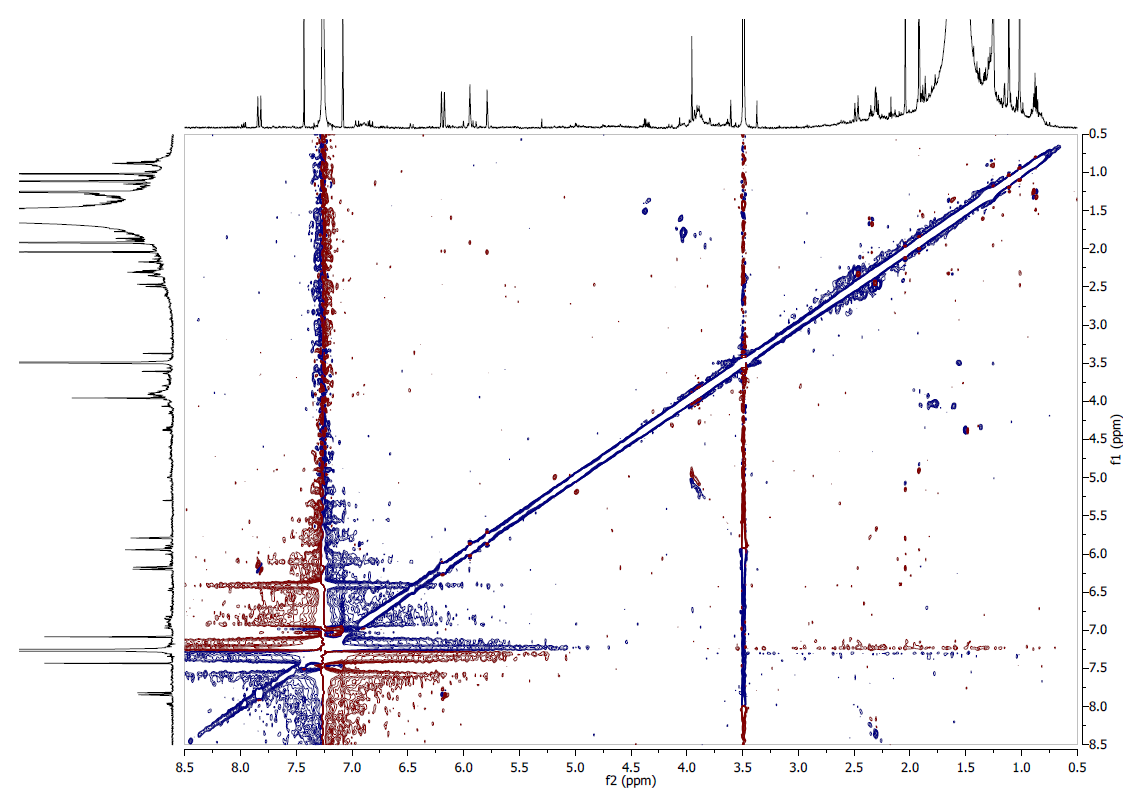


**S15 Fig.** ROESY-NMR spectrum of compound **3** (CDCl_3_)

*
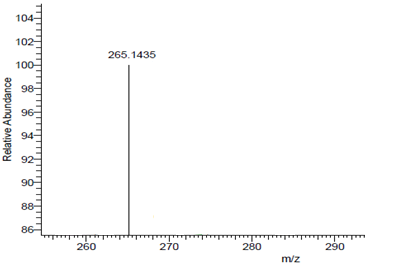
*

**S16 Fig.** HR-ESI-MS spectrum (positive mode) of compound **3**

**

**S17 Fig.** ^1^H-NMR spectrum of compound **4** (600 MHz, CD_3_OD)


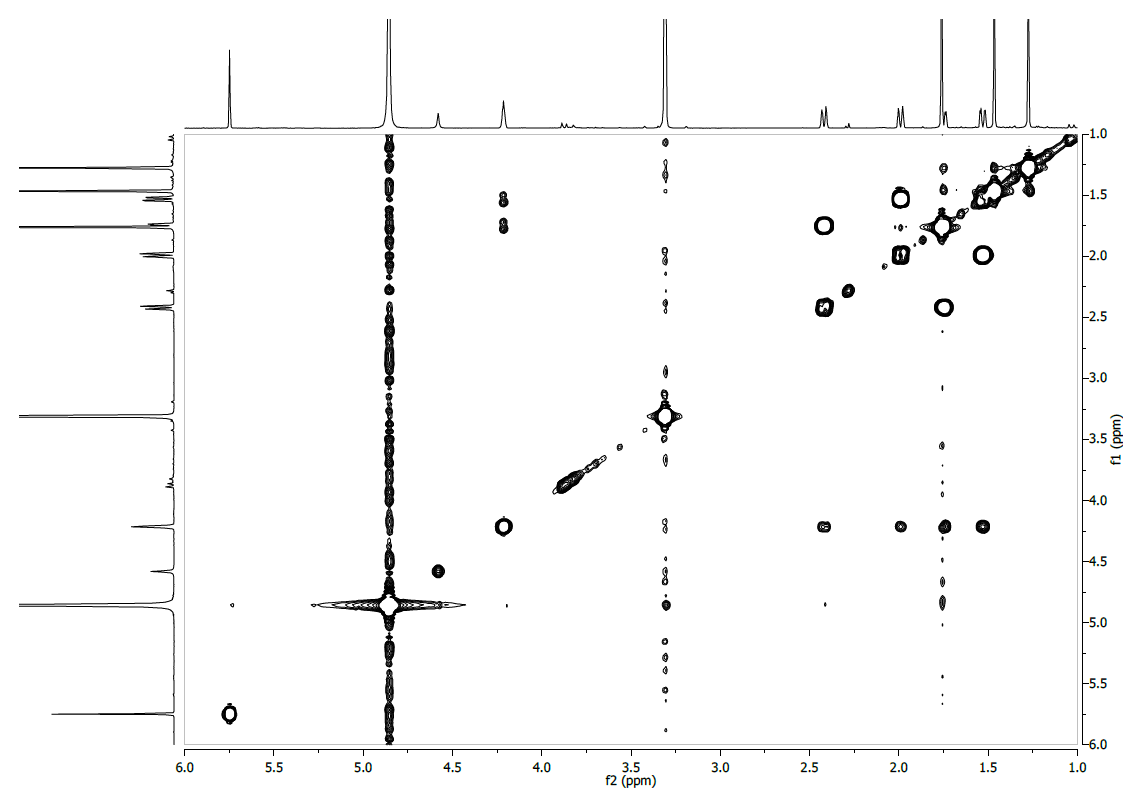


**S18 Fig.** COSY-NMR spectrum of compound **4** (CD_3_OD)

**

**S19 Fig.** DEPTQ-^13^C spectrum of compound **4** (150 MHz, CD_3_OD)


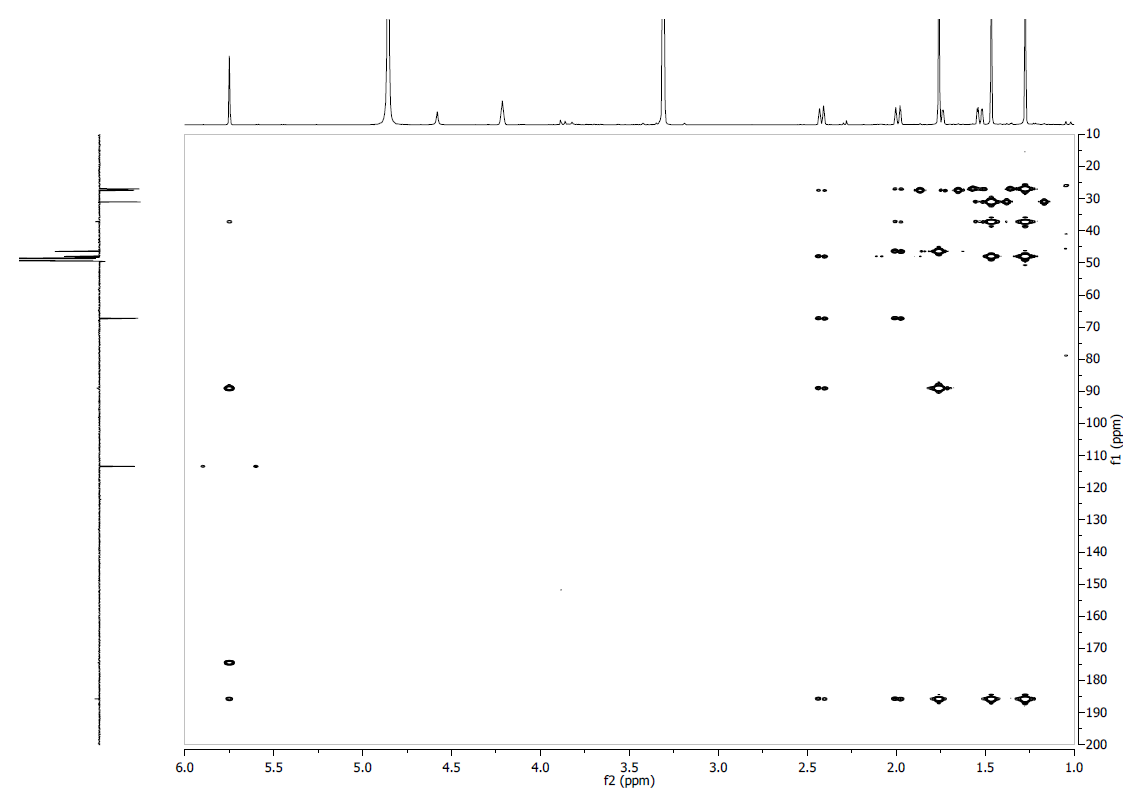


**S20 Fig.** HMBC-NMR spectrum of compound **4** (CD_3_OD)

*
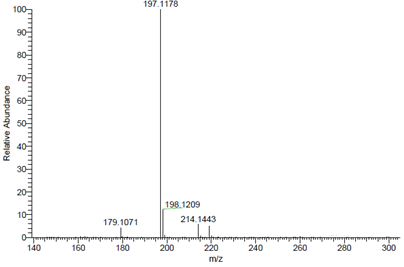
*

**S21 Fig.** HR-ESI-MS spectrum (negative mode) of compound **4**

**S22 Fig.** ^1^H-NMR spectrum of compound **5** (600 MHz, CD_3_OD)

**S23 Fig.** Edited-HSQC-NMR spectrum of compound **5** (CD_3_OD)

**S24 Fig.** HMBC-NMR spectrum of compound **5** (CD_3_OD)


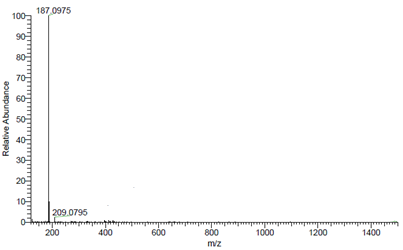


**S25 Fig.** HR-ESI-MS spectrum (negative mode) of compound **5**

**S26 Fig.** ^1^H-NMR spectrum of compound **6** (600 MHz, CD_3_OD)


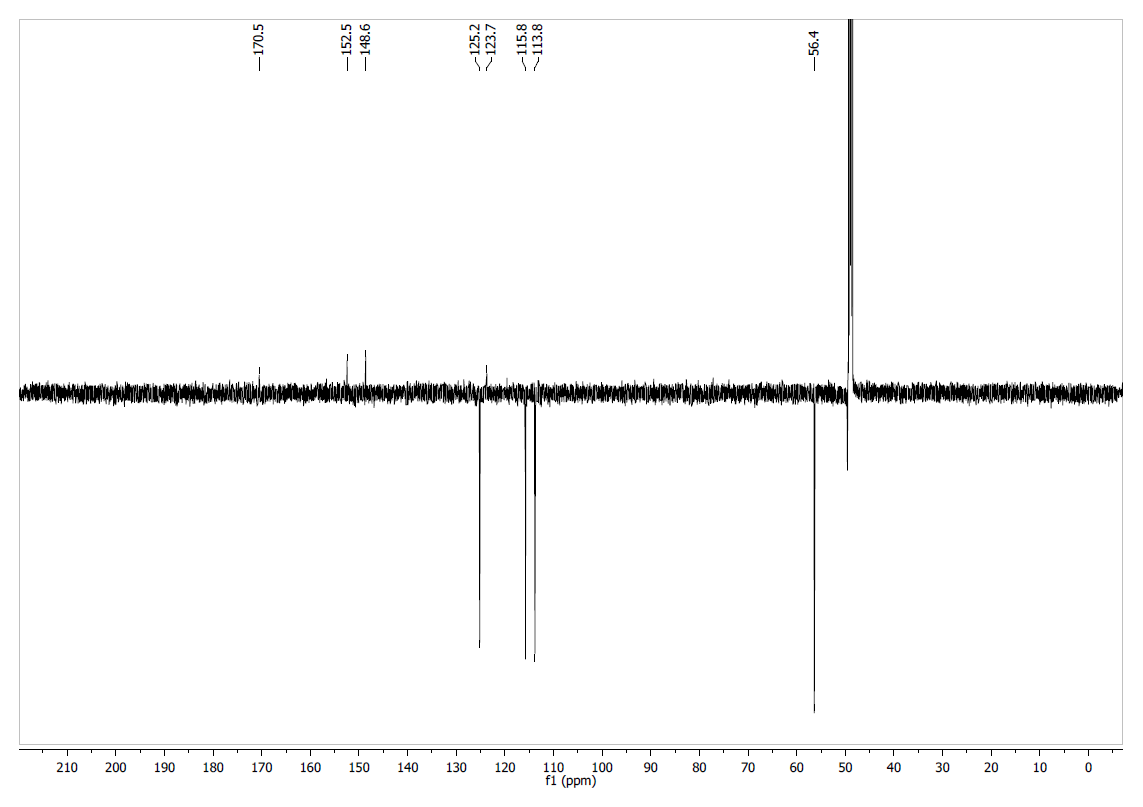


**S27 Fig.** DEPTQ-^13^C-NMR spectrum of compound **6** (150 MHz, CD_3_OD)


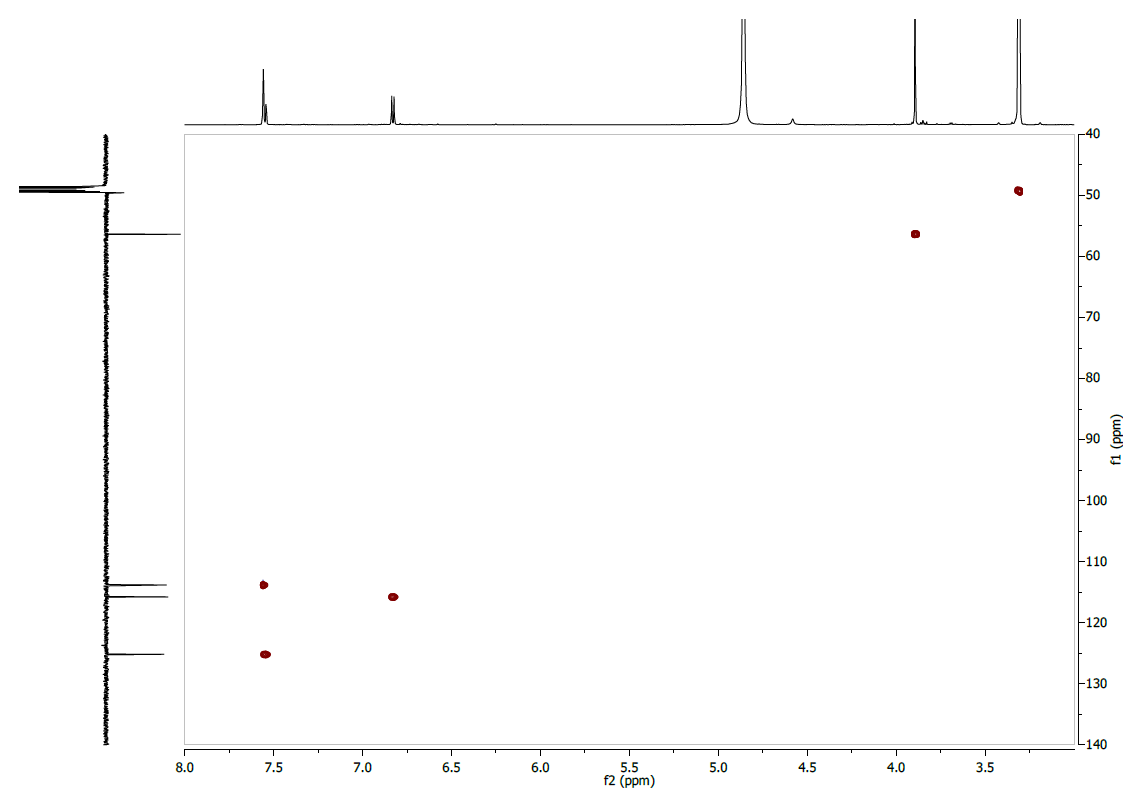


**S28 Fig.** Edited-HSQC-NMR spectrum of compound **6** (CD_3_OD)

**

**S29 Fig.** HMBC-NMR spectrum of compound **6** (CD_3_OD)


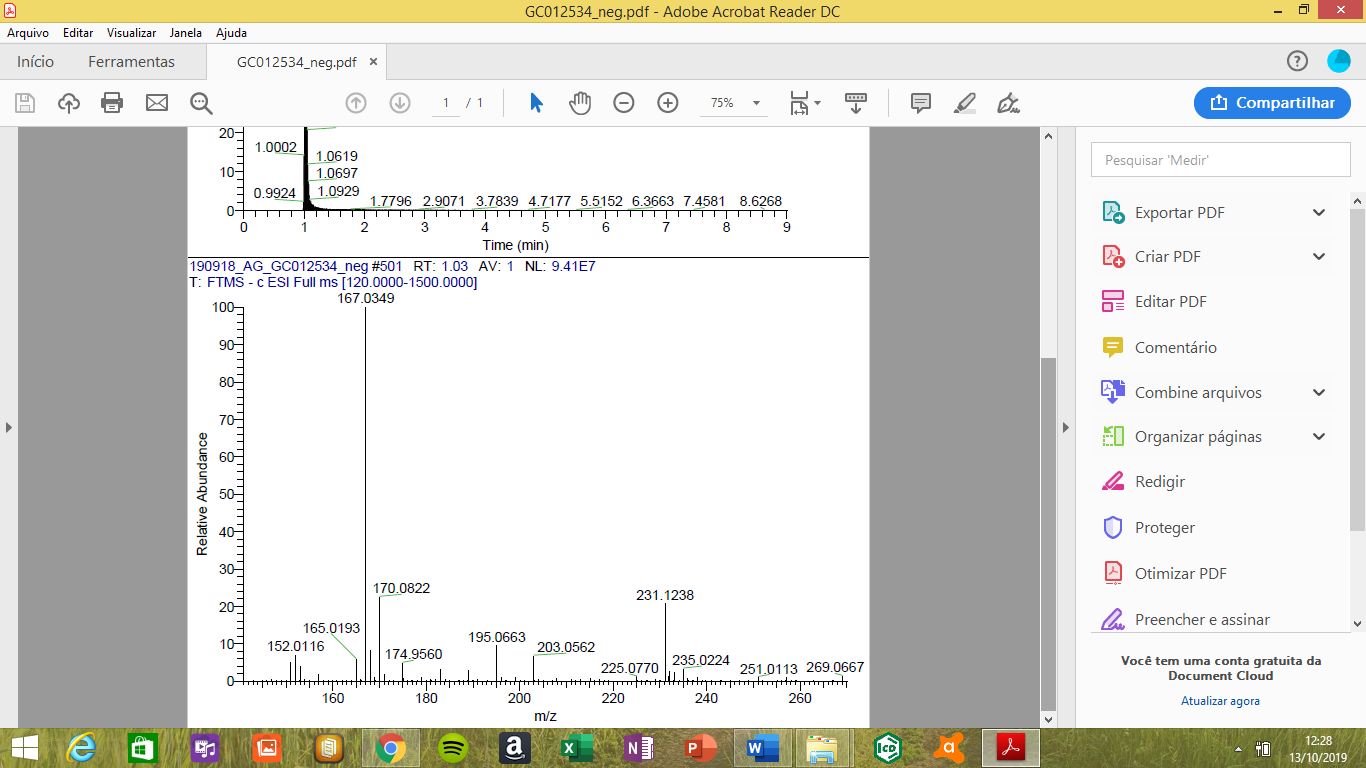


**S30 Fig.** HR-ESI-MS spectrum (negative mode) of compound **6**

**

**S31 Fig.** ^1^H-NMR spectrum of compound **7** (600 MHz, CD_3_OD)

**

**S32 Fig.** Edited-HSQC-NMR spectrum of compound **7** (CD_3_OD)

**

**S33 Fig.** HMBC-NMR spectrum of compound **7** (CD_3_OD)

*
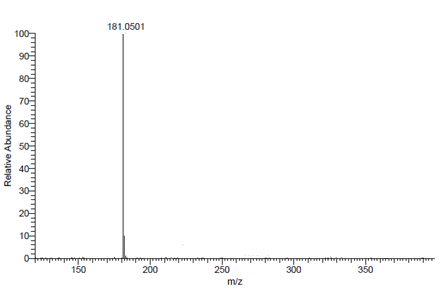
*

**S34 Fig.** HR-ESI-MS spectrum (negative mode) of compound **7**

**

**S35 Fig.** ^1^H-NMR spectrum of compound **8** (600 MHz, CD_3_OD)

**

**S36 Fig.** Edited-HSQC-NMR spectrum of compound **8** (CD_3_OD)

**

**S37 Fig.** HMBC-NMR spectrum of compound **8** (CD_3_OD)

*
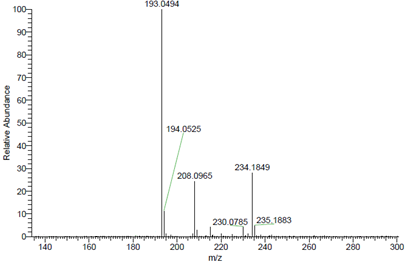
*

**S38 Fig.** HR-ESI-MS spectrum (positive mode) of compound **8**

**S39 Fig.** ^1^H-NMR spectrum of compound **9** (600 MHz, CD_3_OD)


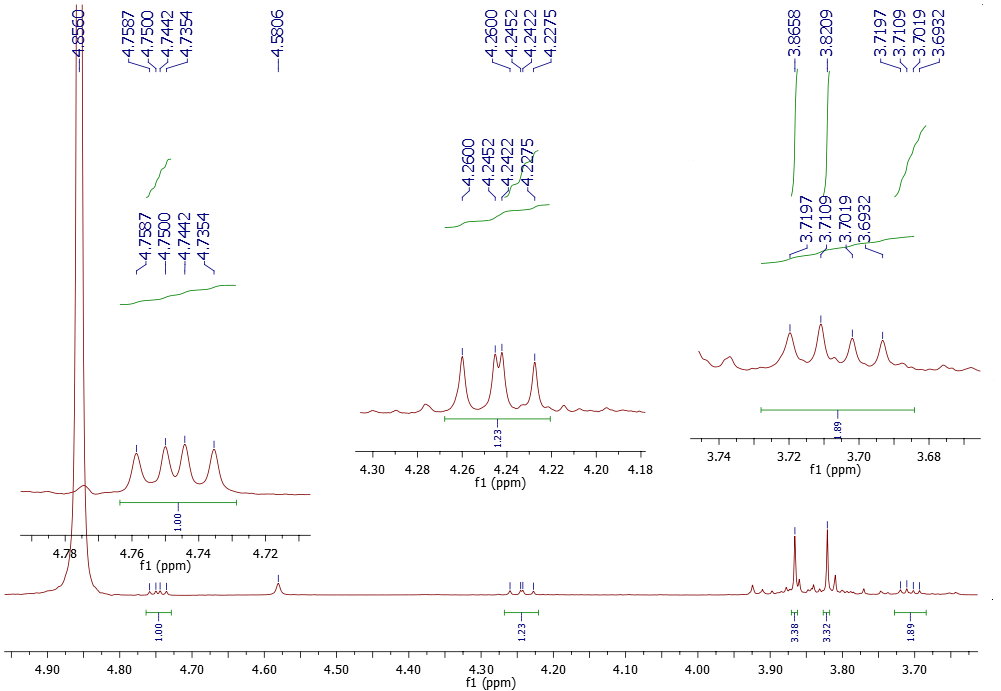


**S40 Fig.** Expansion of ^1^H-NMR spectrum of compound **9** (δ/ppm, 600 MHz, CD_3_OD)

**S41 Fig.** COSY-NMR spectrum of compound **9** (CD_3_OD)

**

**S42 Fig.** Edited-HSQC-NMR spectrum of compound **9** (CD_3_OD)

**

**S43 Fig.** HMBC-NMR spectrum of compound **9** (CD_3_OD)


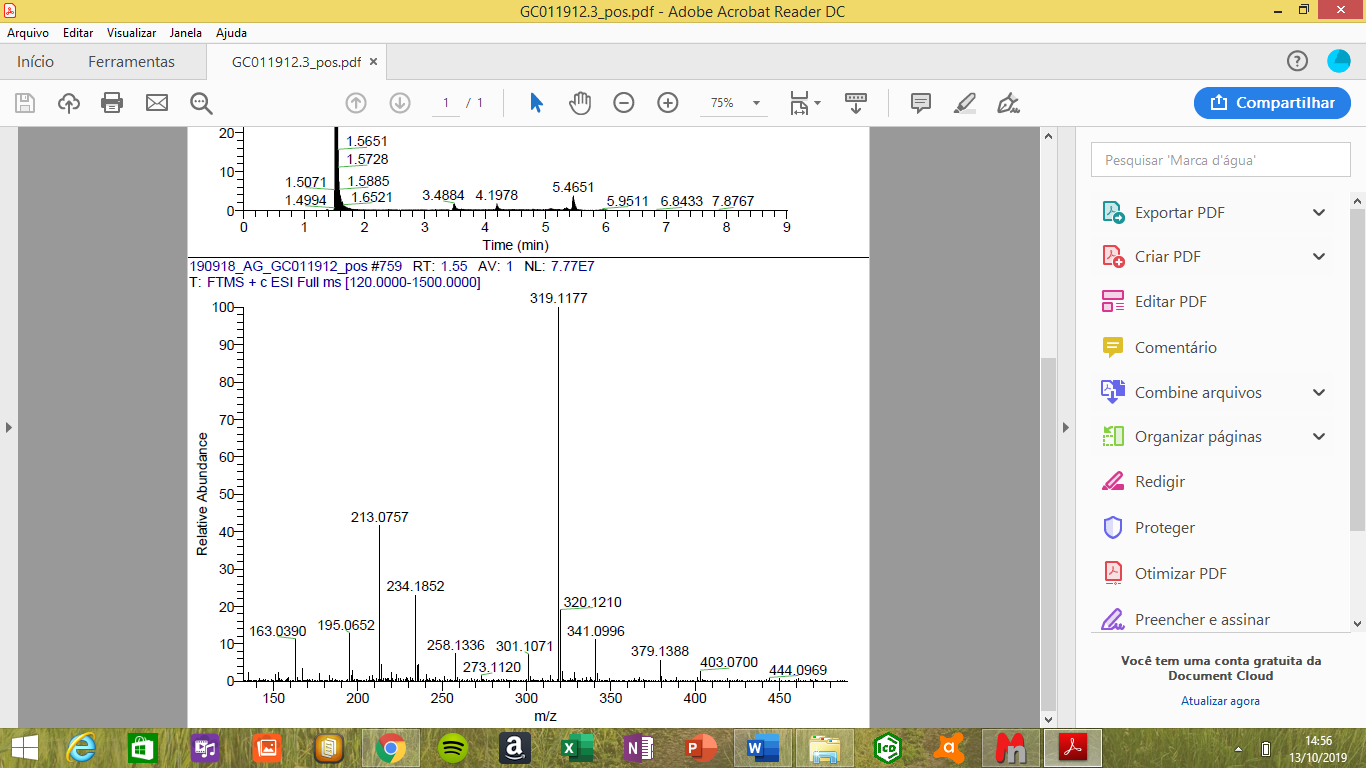


**S44 Fig.** HR-ESI-MS spectrum (positive mode) of compound **9**

**S45 Fig.** ^1^H-NMR spectrum of compound **10** (600 MHz, CD_3_OD)


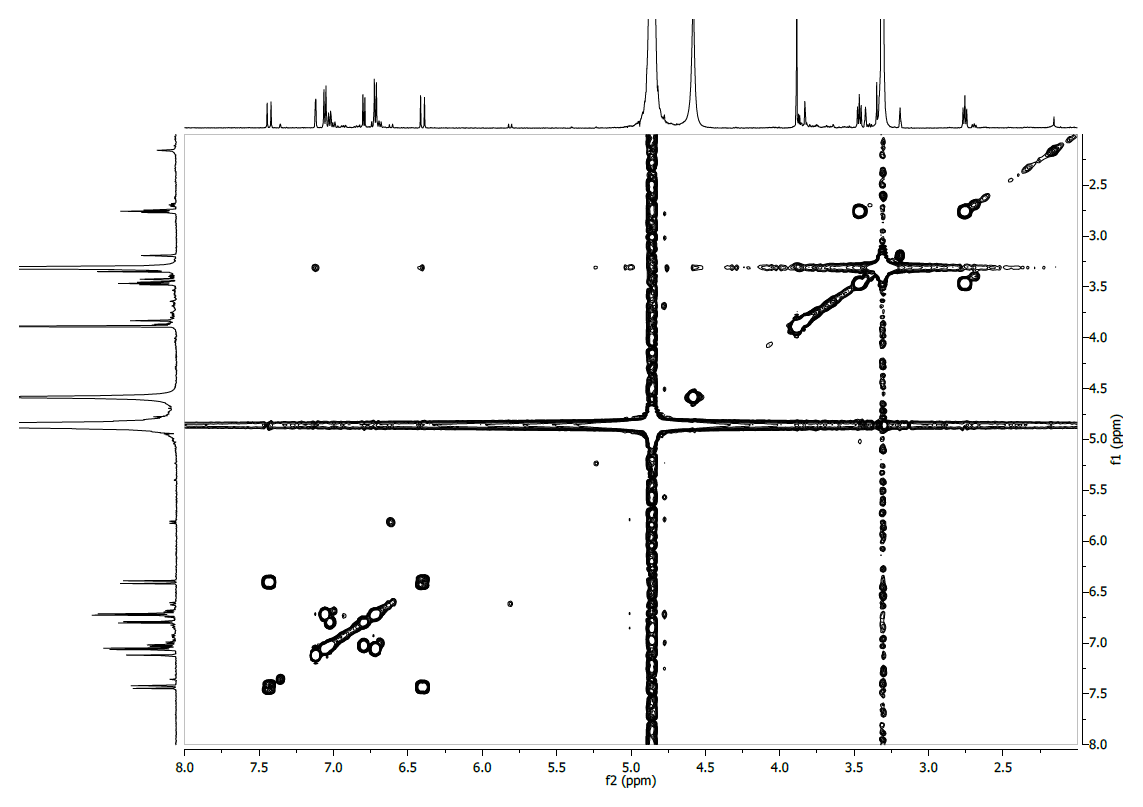


**S46 Fig.** COSY-NMR spectrum of compound **10** (CD_3_OD)

**

**S47 Fig.** HSQC-NMR spectrum of compound **10** (CD_3_OD)

**

**S48 Fig.** HMBC-NMR spectrum of compound **10** (CD_3_OD)


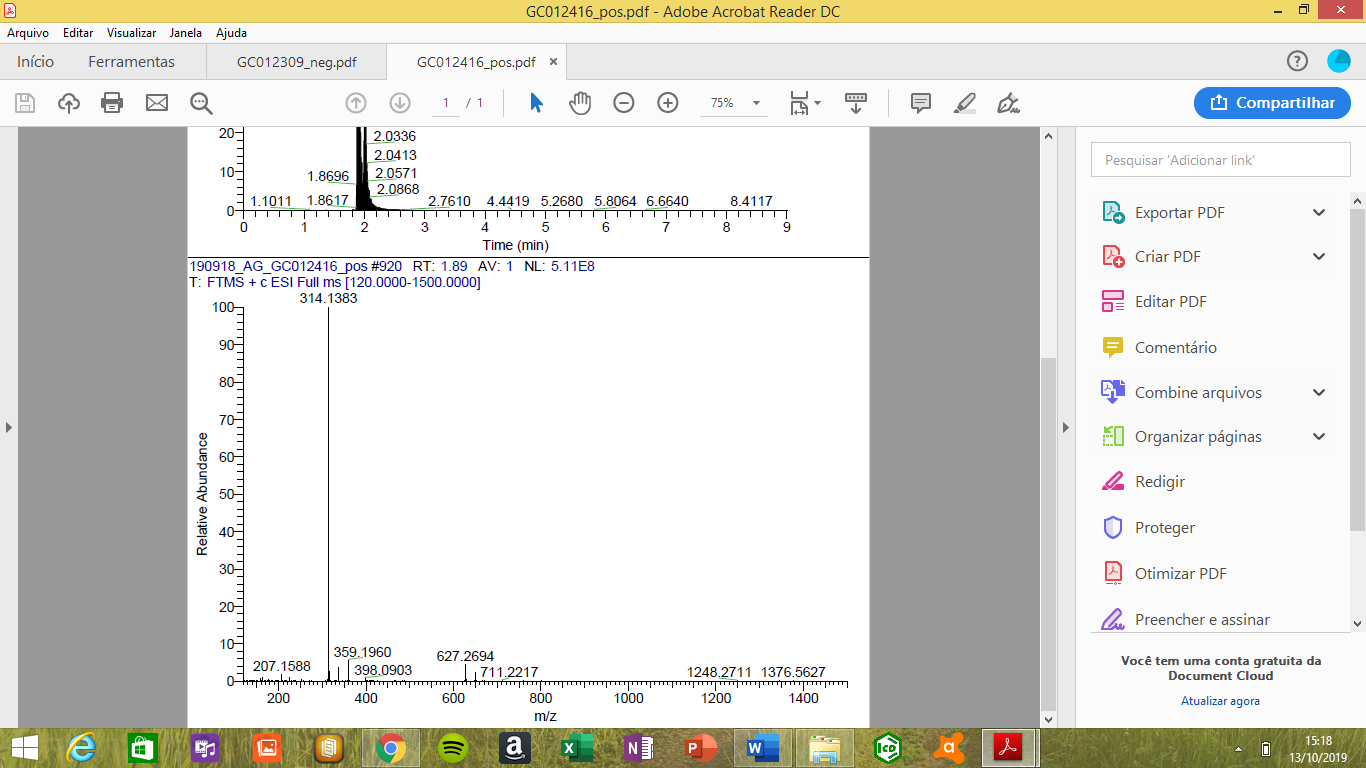


**S49 Fig.** HR-ESI-MS spectrum (positive mode) of compound **10**

**S50 Fig.** ^1^H-NMR spectrum of compound **11** (600 MHz, CD_3_OD)


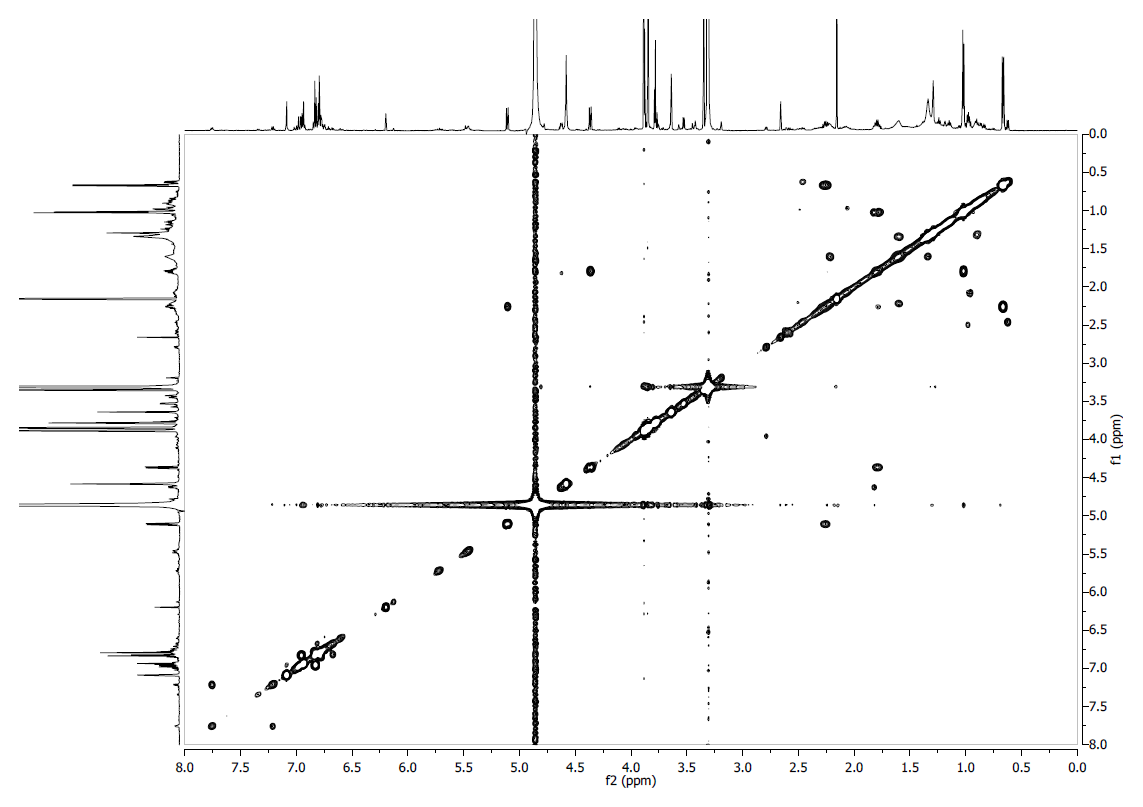


**S51 Fig.** COSY-NMR spectrum of compound **11** (CD_3_OD)

**S52 Fig.** DEPTQ-^13^C spectrum of compound **11** (150 MHz, CD_3_OD)


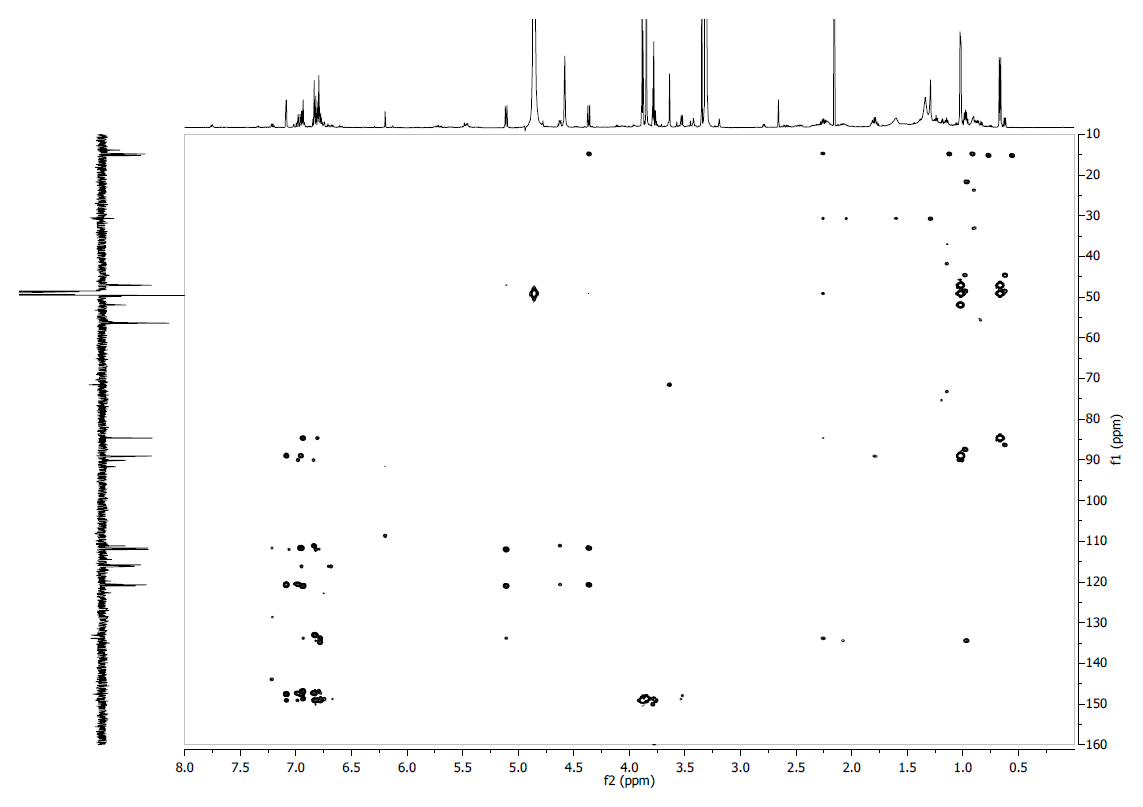


**S53 Fig.** HMBC-NMR spectrum of compound **11** (CD_3_OD)


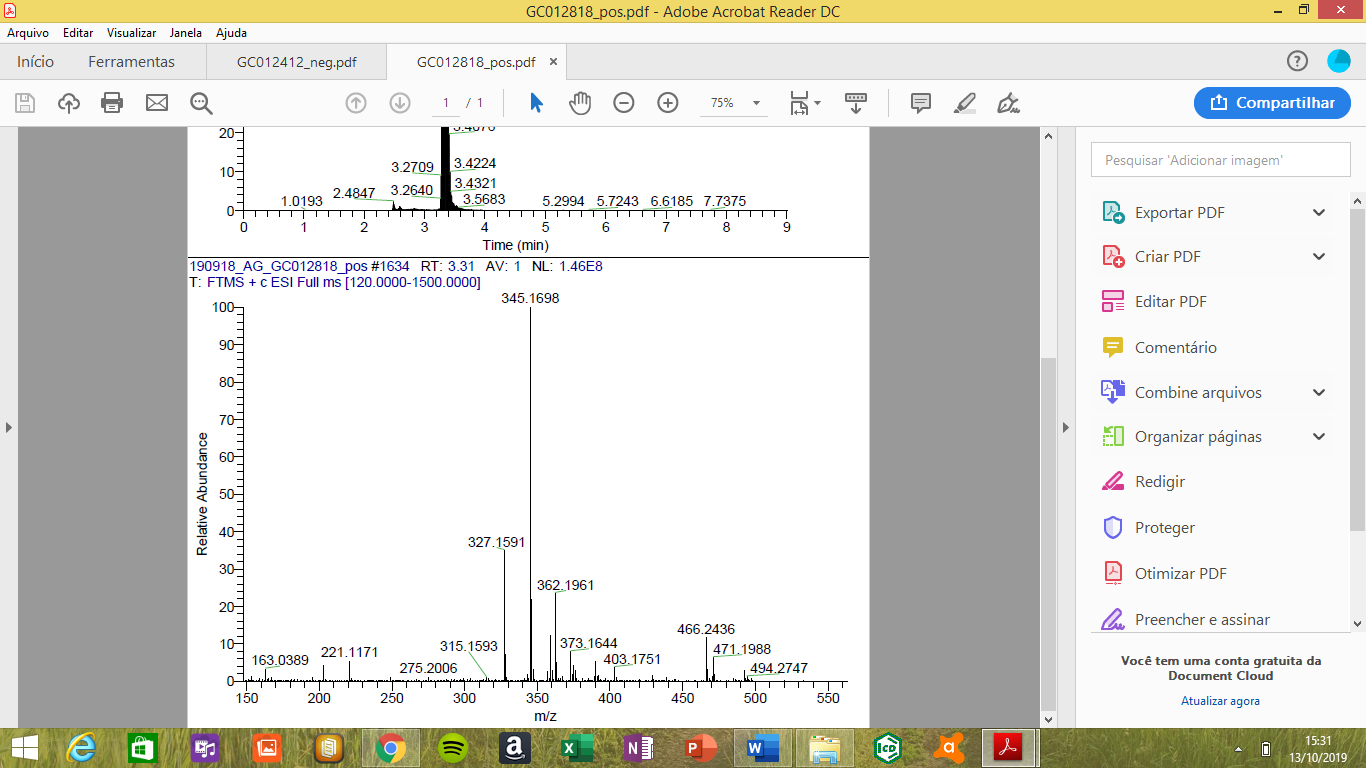


**S54 Fig.** HR-ESI-MS spectrum (positive mode) of compound **11**

**S55 Fig.** ^1^H-NMR spectrum of compound **12** (600 MHz, CD_3_OD)


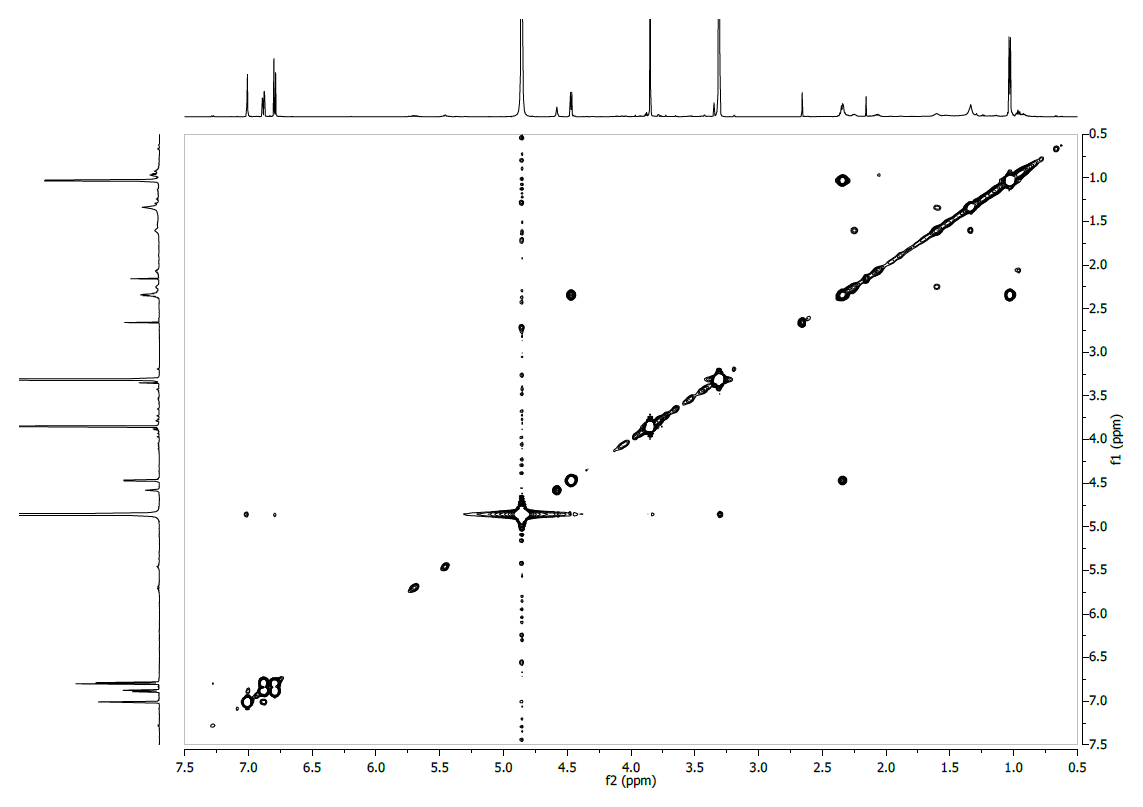


**S56 Fig.** COSY-NMR spectrum of compound **12** (CD_3_OD)


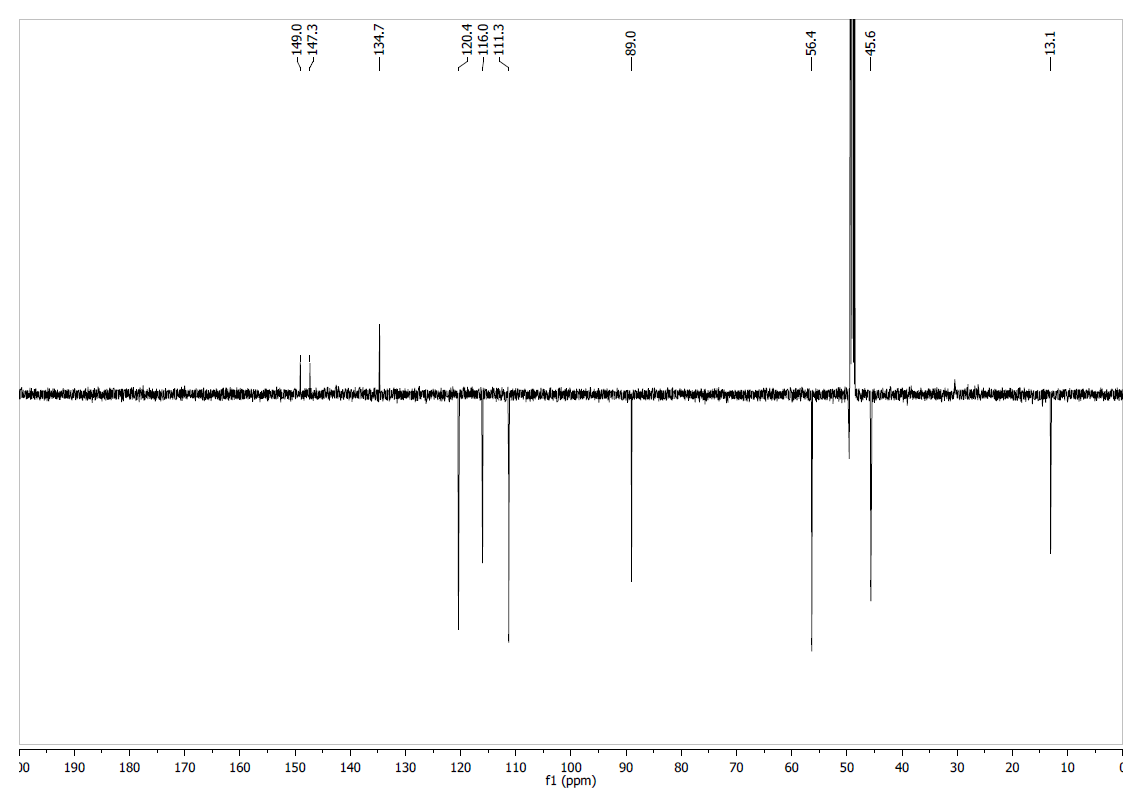


**S57 Fig.** DEPTQ-^13^C-NMR spectrum of compound **12** (150 MHz, CD_3_OD)


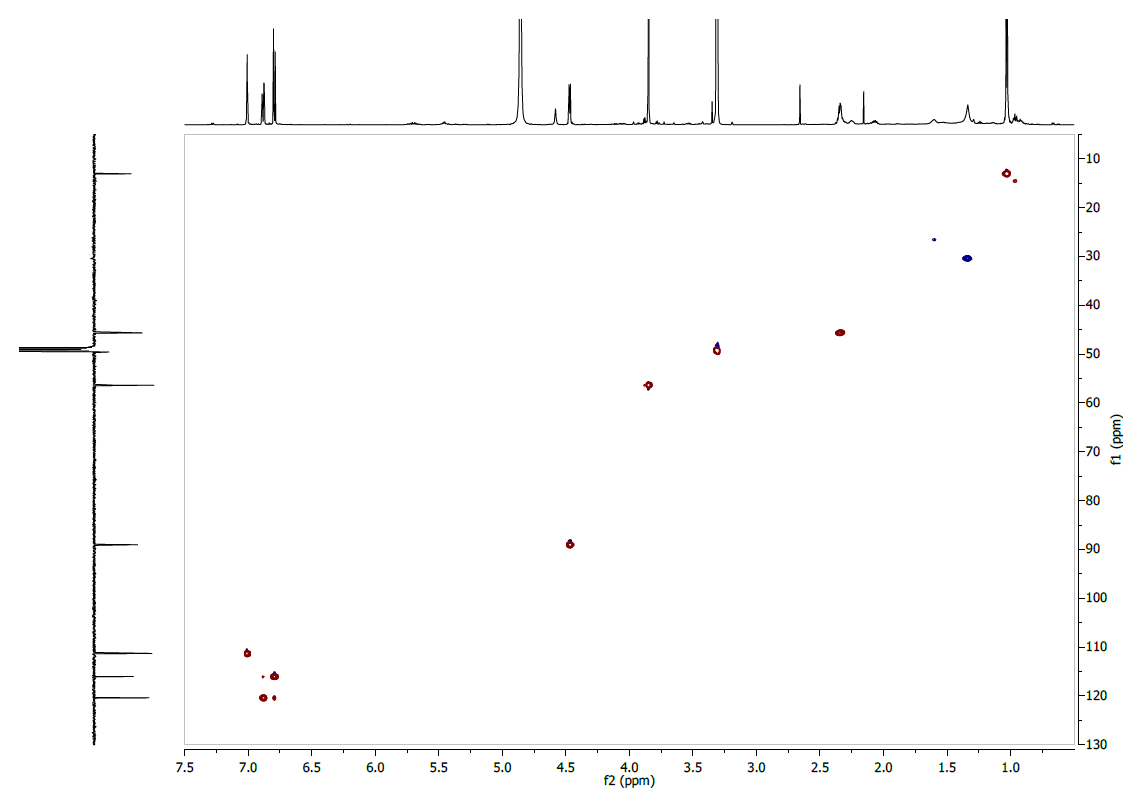


**S58 Fig.** HSQC-NMR spectrum of compound **12** (CD_3_OD)


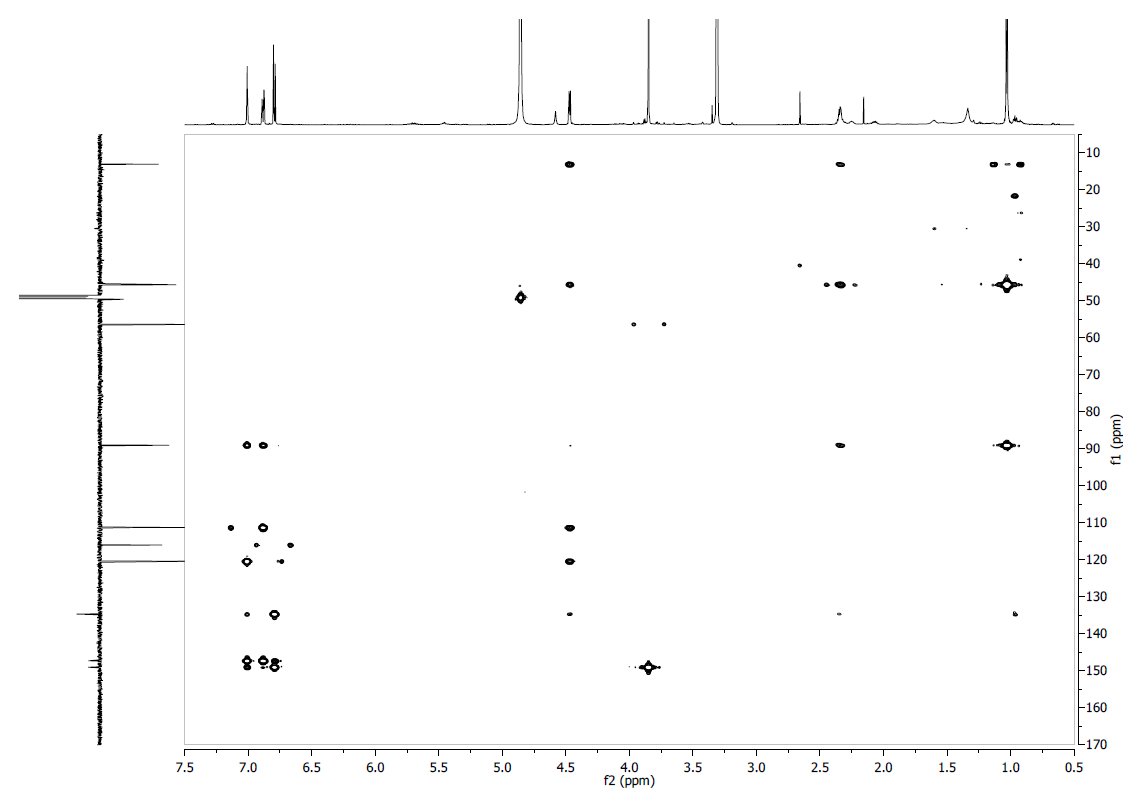


**S59 Fig.** HMBC-NMR spectrum of compound **12** (CD_3_OD)


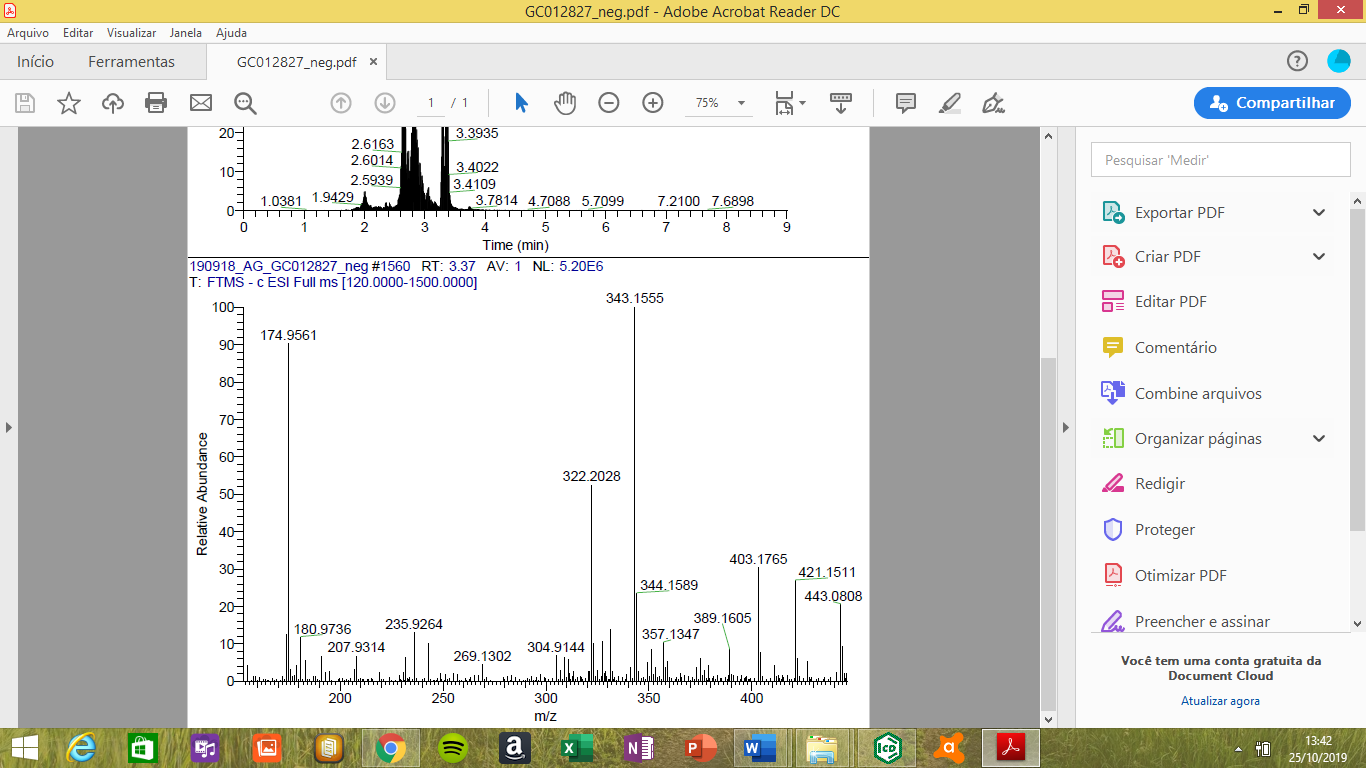


**S60 Fig.** HR-ESI-MS spectrum (negative mode) of compound **12**

SPECTROSCOPIC AND SPECTROMETRIC DATA OF COMPOUNDS **7a – 7e**

*Methyl protocatechuate (****7a****).* Amorphous solid (yield 72%). ESI-HRMS *m/z* 167.0349 [M-H]^-^ (calcd. for C_8_H_7_O_4_, 167.0344). ^13^C NMR (CDCl_3_ + CD_3_OD) δ/ppm: 52.4 (C-2’), 115.6 (C-5), 117.2 (C-2), 123.4 (C-1 and C-6), 146.1 (C-3), 151.8 (C-4), 168.8 (C-1’).

*n-Propyl protocatechuate (****7b****).* Amorphous solid (yield 65%). ESI-HRMS *m/z* 195.0655 [M-H]^-^ (calcd. for C_10_H_11_O_4_, 195.0657). ^13^C NMR (CDCl_3_ + CD_3_OD) δ/ppm: 11.0 (C-4’), 22.3 (C-3’), 66.2 (C-2’), 115.4 (C-5), 117.0 (C-2), 123.2 (C-1 and C-6), 146.0 (C-3), 151.6 (C-4), 168.5 (C-1’).

*n-Butyl protocatechuate (****7c****).* Amorphous solid (yield 61%). ESI-HRMS *m/z* 209.0811 [M-H]^-^ (calcd. for C_11_H_13_O_4_, 209.0814). ^13^C NMR (CDCl_3_ + CD_3_OD) δ/ppm: 13.4 (C-5’), 19.2 (C-4’), 36.3 (C-3’), 64.4 (C-2’), 115.3 (C-5), 117.1 (C-2), 123.2 (C-1 and C-6), 146.0 (C-3), 151.5 (C-4), 168.4 (C-1’).

*n-Pentyl protocatechuate (****7d****).* Amorphous solid (yield 52%). ESI-HRMS *m/z* 223.0978 [M-H]^-^ (calcd. for C_12_H_15_O_4_, 223.0970). ^13^C NMR (CDCl_3_ + CD_3_OD) δ/ppm: 14.0 (C-6’), 22.4 (C-5’), 28.0 (C-4’), 28.5 (C-3’), 64.5 (C-2’), 115.3 (C-5), 117.0 (C-2), 123.2 (C-1 and C-6), 146.0 (C-3),151.4 (C-4), 168.5 (C-1’).

*n-Hexyl protocatechuate (****7e****).* Amorphous solid (yield 41%). ESI-HRMS *m/z* 237.1123 [M-H]^-^ (calcd. for C_13_H_17_O_4_, 237.1126). ^13^C NMR (CDCl_3_ + CD_3_OD) δ/ppm: 14.0 (C-7’), 22.4 (C-6’), 25.2 (C-4’), 31.4 (C-3’), 31.5 (C-5’), 64.5 (C-2’), 115.3 (C-5), 117.0 (C-2), 123.3 (C-1 and C-6), 146.0 (C-3), 151.6 (C-4), 168.5 (C-1’).


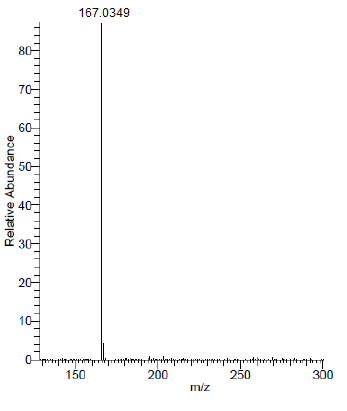


**S61 Fig.** HR-ESIMS spectrum (positive mode) of compound **7a**


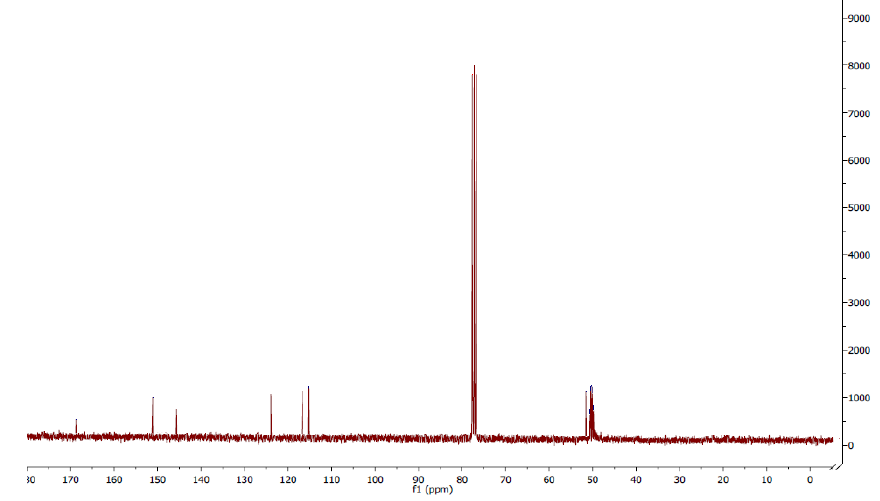


**S62 Fig.** ^13^C NMR spectrum of compound **7a** (CDCl_3_ + CD_3_OD)


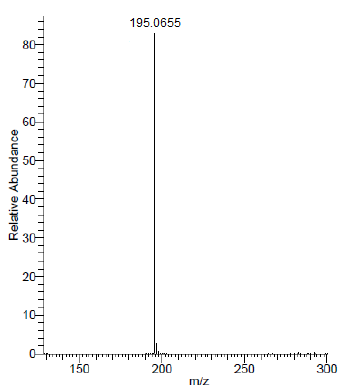


**S63 Fig.** HR-ESIMS spectrum (positive mode) of compound **7b**


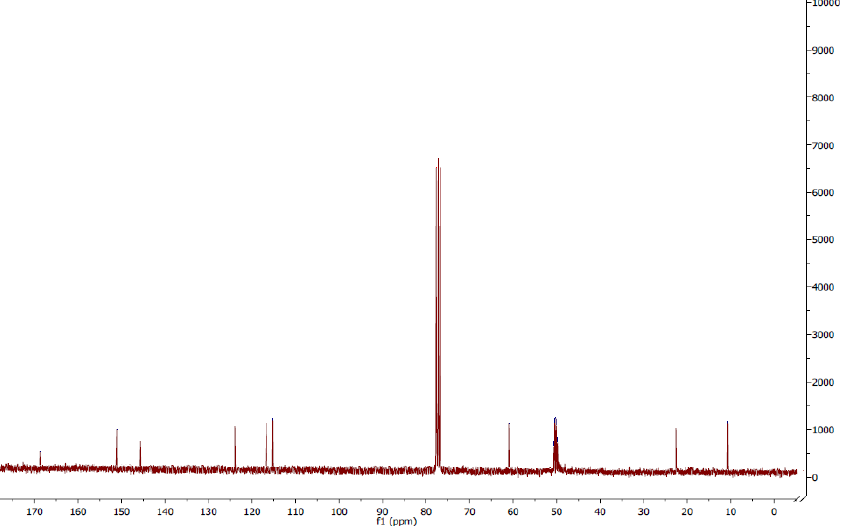


**S64 Fig.** ^13^C NMR spectrum of compound **7b** (CDCl_3_ + CD_3_OD)


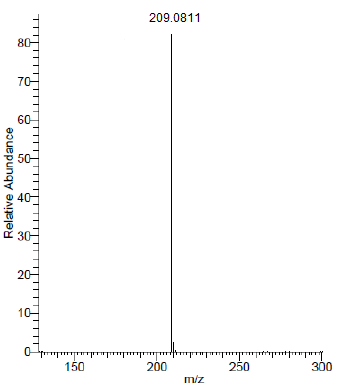


**S65 Fig.** HR-ESIMS spectrum (positive mode) of compound **7c**


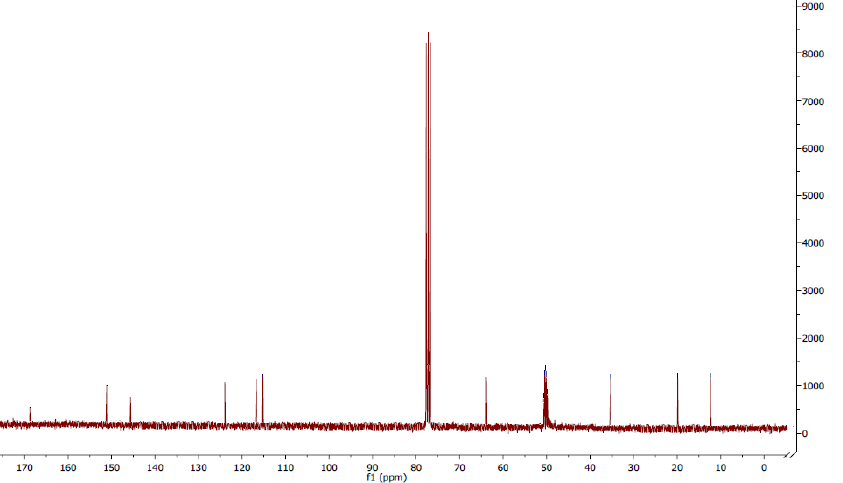


**S66 Fig.** ^13^C NMR spectrum of compound **7c** (CDCl_3_ + CD_3_OD)


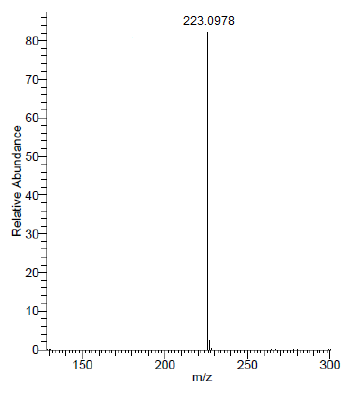


**S67 Fig.** HR-ESIMS spectrum (positive mode) of compound **7d**


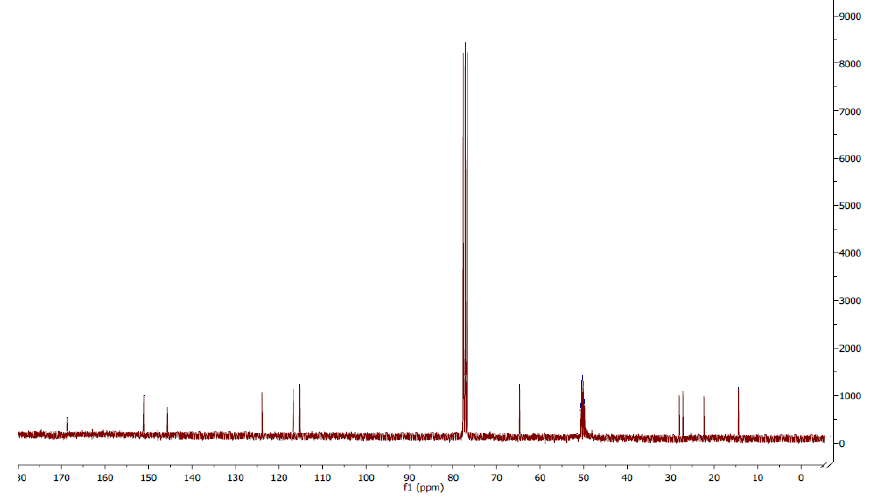


**S68 Fig.** ^13^C NMR spectrum of compound **7d** (CDCl_3_ + CD_3_OD)


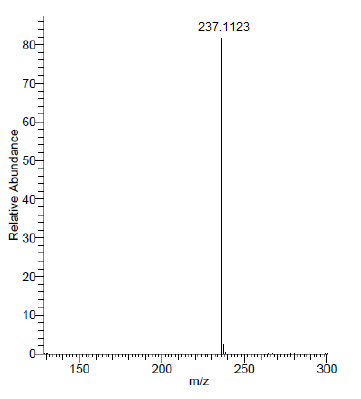


**S69 Fig.** HR-ESIMS spectrum (positive mode) of compound **7e**


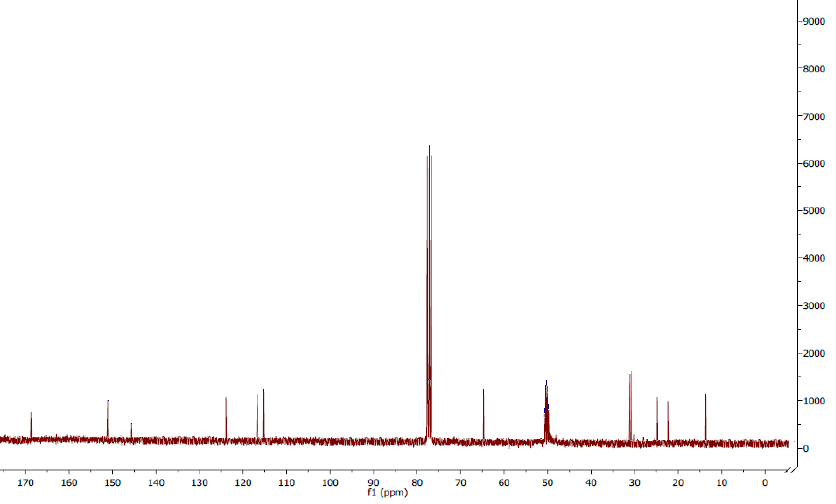


**S70 Fig.** ^13^C NMR spectrum of compound **7e** (CDCl_3_ + CD_3_OD)
