# Supplementary material for: Metabolite profile of Nectandra oppositifolia Nees & Mart. and assessment of antitrypanosomal activity of bioactive compounds through efficiency analyses
Source: PLoS One. 2021 Feb 25;16(2):e0247334. doi: 10.1371/journal.pone.0247334 (PMC7906415; doi:10.1371/journal.pone.0247334)
Supplement: Attachment — Submitted filename: Response to Reviewers.docx [file pone.0247334.s002.docx]

**EDITORIAL OFFICE COMMENTS**

**1. Please ensure that your manuscript meets PLOS ONE's style requirements, including those for file naming. The PLOS ONE style templates can be found at**

**https://journals.plos.org/plosone/s/file?id=wjVg/PLOSOne_formatting_sample_main_body.pdf and**

**https://journals.plos.org/plosone/s/file?id=ba62/PLOSOne_formatting_sample_title_authors_affiliations.pdf**

Thanks for your note – the revised version of this manuscript was adequately revised in order to meet the PLoS ONE style.

**2. We noticed you have some minor occurrence of overlapping text with the following previous publications, which needs to be addressed:**

- [**https://www.sciencedirect.com/science/article/abs/pii/S0045206819315536?via%3Dihub**](https://www.sciencedirect.com/science/article/abs/pii/S0045206819315536?via%3Dihub)
- [**https://onlinelibrary.wiley.com/doi/abs/10.1002/elps.201900240**](https://onlinelibrary.wiley.com/doi/abs/10.1002/elps.201900240)
- [**https://www.mdpi.com/1660-3397/18/1/47/html**](https://www.mdpi.com/1660-3397/18/1/47/html)
- [**https://www.frontiersin.org/articles/10.3389/fpls.2020.01287/full**](https://www.frontiersin.org/articles/10.3389/fpls.2020.01287/full)

Dear editor, thanks for this important note. As observed, these minor overlapping texts refer to Experimental Part, especially bioassays and efficient analysis.

**In your revision ensure you cite all your sources (including your own works), and quote or rephrase any duplicated text.**

The manuscript was carefully revised to avoid duplicated text and the references were adequately cited.

**REVIEW COMMENTS TO THE AUTHOR**

**Reviewer #1**

**Introductive lines on potential of bioactive compounds and nutraceuticals should be added and related references added such as:**

**Durazzo, A.; Lucarini, M.; Santini, A. Nutraceuticals in Human Health. Foods 2020, 9, 370.**

Thanks for your suggestion – this reference and related papers were added in the revised version of this manuscript.

**Lines 68-73 should be enlarged**

This sentence was modified accordingly.

**Data in Table 1 should be better described in the text**

More details concerning the analysis described in Table 1 were included in the revised version of this manuscript.

**Lines 367-373 should be rewritten**

These sentences were rewritten as suggested.

**Lines 484-490 should be better discussed.**

Thank you for this observation. This part of the manuscript was rephrased and the discussion was slightly modified. We hope that these modifications (highlighted in the revised version) had improved the discussion of this topic.

**Major details should be given in Tables 2, 3, 4, 5**

Additional information on Tables 2 – 5 were included as suggested.

**Data in Figures 3 and 4 should be better described in the text**

ClassyFire allows chemists to perform large-scale, rapid and automated chemical classification. Moreover, the accessible API allows easy access to more than 77 million “ClassyFire” classified compounds. The results can be used to help annotate known compounds as performed in the present work. Using this approach, as detailed indicated in figure 3, nineteen different and known compounds (**A – S**) were putatively identified in the studied extracts. Attending your suggestion, one reference was included in this part to allow to reads how these analyses were performed.

In the case of figure 4, additional information was included in the revised manuscript, as suggested by the referee.

**Limits, advantages, practical applications should be added in a paragraph Conclusion**
This sentence was modified accordingly as could be seen in the revised version of manuscript.

**The resolution of all Figures should be improved**

Attending your suggestion, the resolution of all figures was improved as could be seen in the revised version of the manuscript.

**Reviewer #2**

**The paper entitled "Metabolite profile of *Nectandra oppositifolia* and assessment of the antitrypanosomal activity of bioactive compounds through efficiency analyses” describe the dereplication of the bioactive fractions and the isolation of 12 known compounds. Compound 7 was the most active against *T. cruzi*, and derivatives were synthesized and tested.**

**Only few minor modifications are advised for acceptance:**

**1) Line 100, please correct the name of the city: Montluçon.**

Thanks for your careful reading and corrections in the manuscript. This point was modified accordingly in the revised manuscript.

**2) Line 360, please correct and of PI and NI data.**

Thanks again for your careful reading. This point corrected in the revised manuscript.

**3) The quality of the figures 3 and 4 need to be improved.**

The quality of all figures was improved attending your suggestion.

**4) Please add the position numbering on the structures to match the NMR data.**

Attending your suggestion, the structures of compounds **1 – 12** as well as the semisynthetic derivatives **7a – 7e**, containing the position numbering, were included in the Supplementary Material.
